# Hyaluronan (HA)-inspired glycopolymers as molecular tools for studying HA functions†

**DOI:** 10.1039/d0cb00223b

**Published:** 2021-01-28

**Authors:** Dominic W. P. Collis, Gokhan Yilmaz, Yichen Yuan, Alessandra Monaco, Guy Ochbaum, Yejiao Shi, Clare O’Malley, Veselina Uzunova, Richard Napier, Ronit Bitton, C. Remzi Becer, Helena S. Azevedo

**Affiliations:** School of Engineering and Materials Science, Queen Mary University of London London E1 4NS UK h.azevedo@qmul.ac.uk; Department of Chemistry, University of Warwick CV4 7AL UK Remzi.Becer@warwick.ac.uk; Department of Chemical Engineering and the Ilza Katz, Institute for Nanoscale Science & Technology, Ben-Gurion University of the Negev Beer-Sheva 84105 Israel; Institute of Bioengineering, Queen Mary University of London London E1 4NS UK; School of Life Sciences, University of Warwick CV4 7AL UK

## Abstract

Hyaluronic acid (HA), the only non-sulphated glycosaminoglycan, serves numerous structural and biological functions in the human body, from providing viscoelasticity in tissues to creating hydrated environments for cell migration and proliferation. HA is also involved in the regulation of morphogenesis, inflammation and tumorigenesis through interactions with specific HA-binding proteins. Whilst the physicochemical and biological properties of HA have been widely studied for decades, the exact mechanisms by which HA exerts its multiple functions are not completely understood. Glycopolymers offer a simple and precise synthetic platform for the preparation of glycan analogues, being an alternative to the demanding synthetic chemical glycosylation. A library of homo, statistical and alternating HA glycopolymers were synthesised by reversible addition–fragmentation chain transfer polymerisation and post-modification utilising copper alkyne–azide cycloaddition to graft orthogonal pendant HA monosaccharides (*N*-acetyl glucosamine: GlcNAc and glucuronic acid: GlcA) onto the polymer. Using surface plasmon resonance, the binding of the glycopolymers to known HA-binding peptides and proteins (CD44, hyaluronidase) was assessed and compared to carbohydrate-binding proteins (lectins). These studies revealed potential structure-binding relationships between HA monosaccharides and HA receptors and novel HA binders, such as Dectin-1 and DEC-205 lectins. The inhibitory effect of HA glycopolymers on hyaluronidase (HAase) activity was also investigated suggesting GlcNAc- and GlcA-based glycopolymers as potential HAase inhibitors.

## Abstract 2

Glycopolymers based on hyaluronan (HA) were synthesised as homopolymers and alternating copolymers and used as probes to study the interactions with known HA-binding proteins and peptides.

## Graphical abstract

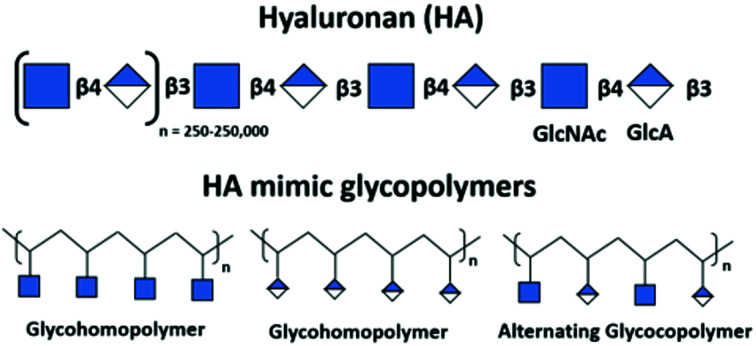

## Introduction

Hyaluronic acid (HA) is a unique member of the glycosaminoglycan (GAG) family^1^ which are linear polysaccharides containing a core repeating disaccharide unit. The HA disaccharide consists of *N*-acetyl-d-glucosamine (GlcNAc) and glucuronic acid (GlcA) connected by β(1–4) and β(1–3) glycosidic bonds in an alternating fashion and forming long chains in the mega-Dalton range, far surpassing the length of any other GAGs. Unlike other GAGs, HA is non-sulfonated and does not form covalent bonds with the protein core in proteoglycans. Instead, HA binds to specific proteins known as hyaladherins. Examples of HA-binding proteins (HABPs) are cell surface receptors, such as the Cluster of Differentiation 44 (CD44), or matrix proteins like link protein (LP) that connects HA to aggrecan in cartilage extracellular matrix. HA has been exploited in many biomedical applications including areas such as ophthalmology, orthopaedics and drug delivery.^2^ Currently, ultrapure HA is still obtained by extraction from natural sources,^3^ such as rooster combs, bovine vitreous humour and also from engineered bacteria.^4^ The chemical synthesis of this large polysaccharide with precision and control would be challenging and has never been attempted.

Glycopolymers provide a simpler alternative method to the total chemical synthesis of glycans (*e.g.* synthesis of precisely defined oligosaccharides by chemical glycosylation). They provide two key components: the sugar moieties, which are biologically active and recognised by specific proteins, and the stability of a polymer backbone. Despite the lack of glycosidic bond, glycopolymers have been shown to be recognized by carbohydrate-binding domains in proteins and have been exploited in areas such as enzyme inhibition.^5–7^ Many carbohydrate receptors are located on the cell surface, allowing the reversible binding of polymers to multiple receptors. With some receptors inducing signal transduction upon binding, this weak signal is amplified as multiple receptors are triggered simultaneously. The multivalency of glycopolymers permits the formation of strong interactions with receptors which can be used to dissect the role of structure and function of natural glycans.^8^

Advances in synthetic polymerization techniques in the past two decades allow precise control over the polymerisation process. Currently, polymers can be produced with high precision for dispersity (*Đ*), degree of polymerisation^9,10^ (DP) and sequence^11,12^ through controlled radical polymerisation methods, such as reversible addition–fragmentation chain transfer (RAFT),^13,14^ atom transfer radical polymerisation (ATRP)^15^ and single electron transfer living radical polymerisation (SET-LRP).^16^ The synthesis of glycopolymers has been achieved through click chemistry^17^*via* regioselective and chemoselective reactions to link the saccharide units to either a monomer, for pre-polymerisation modification, or orthogonally post-polymerisation.^18,19^ With a large array of polymerisation methods, monomers and chemoselective reactions available, more complex glycopolymers are being designed and synthesized.^20,21^

GAG mimetic polymers have been previously synthesised by methods such as ring-opening metathesis polymerisation (ROMP) and step-growth polymerisations to form HA-,^22^ heparan sulfate-(HS)^23,24^ and chondroitin sulfate-(CS)^25,26^ based glycopolymers. However, the synthesis of such glycopolymers involves challenging multistep reactions resulting in low yields to produce a disaccharide with a single glycosidic bond. Herein, glycopolymers bearing single or alternating HA monosaccharides have been synthesised and used as synthetic tools to dissect the binding of HA to known and new proteins and as potential HA-mimetic therapeutics.

## Results and discussion

### Synthesis and characterisation of glycopolymers

**Scheme 1:**
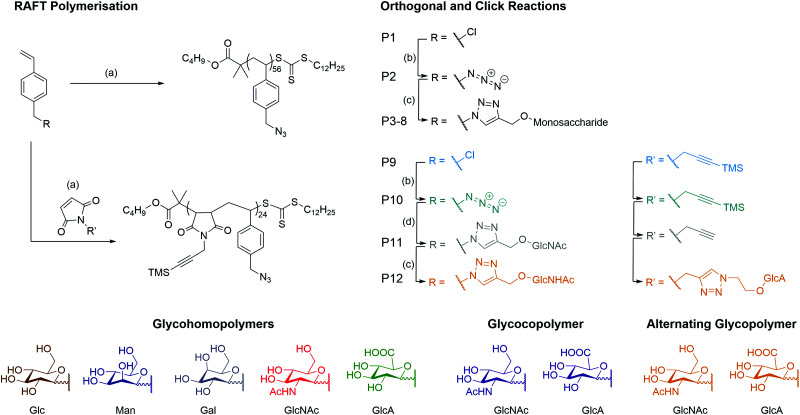
Synthetic overview of the pathways for the formation of the homo- and alternating glycopolymers. Reagents and conditions: (a) V601, CTA, dioxane, 70 °C, 16 h (b) NaN_3_, DMSO, 16 h (c) Cu(i)Br, Me_6_Tren, DMSO, 24 h (d) (i) Cu(i)Br, Me_6_Tren, DMSO, 24 h (ii) K_2_CO_3_, H_2_O.

A series of monosaccharides were synthesised (Scheme S1, ESI†) to display a click moiety at the anomeric centre. This was achieved by direct glycosylation with an alcohol donor using H_2_SO_4_–silica.^27^ However, for GlcA, the monosaccharide requires acetylation followed by esterification to inhibit the formation of a bicyclic lactone,^28^ before using BF_3_·OEt_2_ as a catalyst to form the glycosidic bond with the alcohol donor (Scheme S2, ESI†). The glycopolymers were then synthesised by first producing a well-defined polymer backbone of poly(4-vinylbenzyl chloride) (**P1**) using RAFT polymerisation to give **P1** with a dispersity (*Đ*) of 1.13 (Scheme 1 and Table 1). The pendant chloride was converted to azide (**P2**) and then ‘clicked’ with a monosaccharide alkyne by copper alkyne–azide cycloaddition (CuAAc), to produce glycohomopolymers (**P3–P7**) bearing different sugar monomers (Glc, Man, Gal, GlcNAc, GlcA) and the statistical glycocopolymer (**P8**). The formation of the glycopolymers was monitored by FT-IR (Fig. S2, ESI†), GPC (Fig. S3, ESI†) and ^1^H NMR (Fig. S5, ESI†).

**Table 1:** Characterisation of the polymerisation and relevant properties of the glycopolymers prepared in this study

	Polymer	Type	*M*_n_ (g mol^−1^)		*Đ* b
			Theo^a^	GPC^b^	
**P1**	pVBC_56_	Homopolymer	12 800	13 100	1.13
**P2**	pVBAz_56_	Homopolymer	13 400	13 400	1.20
**P3**	pVB-Glc_56_	Glycohomopolymer	32 000	4400	1.07
**P4**	pVB-Man_56_	Glycohomopolymer	32 000	4400	1.08
**P5**	pVB-Gal_56_	Glycohomopolymer	32 000	4300	1.04
**P6**	pVB-GlcNAc_56_	Glycohomopolymer	36 000	6900	1.13
**P7**	pVB-GlcA_56_	Glycohomopolymer	35 000	6300	1.02
**P8**	pVB-GlcA_23_-*co*-VB-GlcNAc_23_	Statistical glycocopolymer	35 500	4100	1.04
**P9**	pVBC_24_-*alt*-TMS PMI_24_	Alternating copolymer	12 800	19 500	1.57
**P10**	pVBAz_24_-*alt*-TMS PMI_24_	Alternating copolymer	19 900	18 300	2.40
**P11**	pVB-GlcNAc_24_-*alt*-PMI_24_	Alternating glycocopolymer	27 000	4800	1.02
**P12**	pVB-GlcNAc_24_-*alt*-PMI-GlcA_24_	Alternating glycocopolymer	40 000	5700	1.06

a Determined by NMR conversion of each monomer plus the mass of the RAFT agent.

b Determined by GPC using NH_4_BF_4_ (5 mM) in DMF with PS calibration. *M*_n_ = Number average molecular weight. *Đ* = dispersity.

It was noted that during the azidation, a high molecular weight shoulder appeared in the GPC with peak maximum molar mass (*M*_p_) double that of the main peaks *M*_p_ (Fig. S4, ESI†). The cleavage of RAFT end group by sodium azide has previously been reported in the literature.^29^ Attempts to cleave the RAFT end group^30,31^ were ineffective due to the composition of the polymer, inhibiting some cleavage techniques (*e.g.* styrenic backbone and pendant chloride) or provided only partial cleavage detected by UV detector of the GPC (Fig. S7, ESI†).

Using the styrene-maleic anhydride alternating copolymer system,^2,32,33^ a co-monomer of 1-(3-(trimethylsilyl)prop-2ynyl)-1*H*-pyrrole-2,5-dione (TMS PMI) was produced using the Mitsunobu reaction^33,34^ and added into the RAFT polymerisation medium of VBC (Fig. S9, ESI†). To ensure the alternation in the co-monomer composition, the TMS PMI was added in excess to reduce the potential of VBC block formation within the copolymer. The TMS group is vital for the polymerisation of the maleimide monomer as it prevents the crosslinking of chains through the alkyne (Fig. S8, ESI†) and inhibition of intra or intermolecular cyclisation of **P10** during the first set of the CuAAC reactions.

**Fig. 1:**
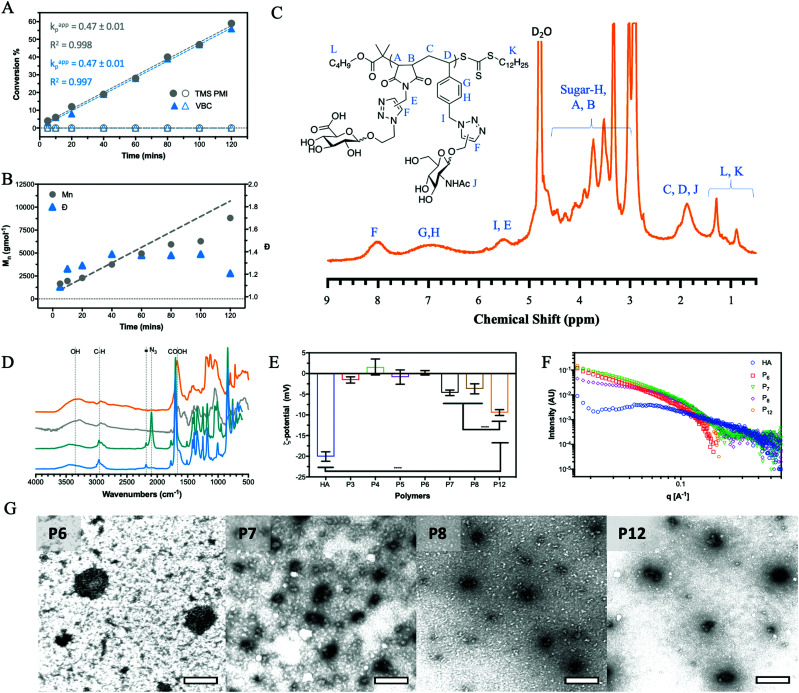
Chemical and structural characterization of synthesized glycopolymers. (A) Kinetics of the two monomers comparing them individually (hollow) and in a copolymerisation (solid) with (B) control over the polymerisation using RAFT. The resulting polymer is converted to the final alternating glycopolymer, **P12** (orange) as shown by (C) ^1^H NMR with the reactions being monitored by (D) FT-IR following the conversion of the functional groups (**P9**, light blue; **P10**, teal; **P11** grey; **P12**, orange). (E) Zeta-potential at 100 μM in H_2_O at pH 7.0, (F) SAXS of the glycopolymers and HA at 200 μM in H_2_O at pH 7.0 along with (G) TEM images of the aggregates formed by the HA glycopolymers at 1 mM in H_2_O at pH 7.0 and stained with uranyl acetate (scale bar 1 μm). Analysed by 2way ANOVA analysis, **** significant at *P* < 0.0001.

The alternation was measured by following the polymerisation kinetics (Fig. 1A) which showed that over the initial 2 hours, individual polymerisation of VBC and TMS PMI displayed no conversion. Extended reaction times produced pVBC, while TMS PMI showed no conversion. In comparison, a copolymerisation system showed increased polymerisation rate (*k*_p_) to the homopolymers, that were identical to each other. Owing to the inability of TMS PMI to self-propagate, TMS PMI propagates after each addition of VBC monomer. The stoichiometric excess of TMS PMI, along with the identical *k*_p_ values, indicates a high degree of alternation. The kinetics also showed good control over the growing polymer (Fig. 1B) in both molecular weight (*M*_n_) and dispersity (*Đ*). Furthermore, Matrix-Assisted Laser Desorption Ionization Time-of-Flight Mass Spectrometry (MALDI-ToF-MS) was performed to detect the alternating nature of the copolymer. Whilst the resolution was limited, individual distributions could be assigned with the major distribution with a delta of 360 Da, equivalent to the molecular weight sum of both monomers (Fig. S1, ESI†). The smaller distributions can be assigned as polymers being initiated by the radical initiator or by the RAFT agent with initiation or termination of the polymer with either TMS-PMI or VBC. Several of the peaks have a secondary peak 32 Da away, this arises from the ring-opening of the maleimide by methanol during precipitation.

By a similar methodology used for the homoglycopolymers, the pendant chloride was converted into an azide (**P10**) and then ‘clicked’ with a GlcNAc-alkyne by CuAAc to produce an alternating glycocopolymer. The TMS group can be efficiently cleaved by supplementing the dialysis with K_2_CO_3_.

After which point the resulting polymer (**P11**) can undergo CuAAc with GlcA-azide to form the final alternating glycocopolymer (**P12**). The CuAAc click reaction was monitored by NMR (Fig. 1C), FT-IR (Fig. 1D) and GPC (Fig. S9, ESI†).

### Structural and physical properties of HA glycopolymers

All glycopolymers possess a hydrophobic polymer backbone with orthogonal hydrophilic sugars. Similar glycopolymer systems have shown to aggregate at low concentration due to collapse of the hydrophobic polymer backbone.^35^ To assess the aggregation behaviour of the glycopolymers in water, fluorescence measurements were conducted using Nile red as a fluorescent reporter. Nile red is a sovatochromic dye that has very weak fluorescence intensity in pure water, with an emission maximum at around 660 nm, but when encapsulated in a hydrophobic environment, it emits strong fluorescence at around 620 nm (blue shift).^36^ All the polymers showed a blue shift and an increase in fluorescent output (Fig. S10, ESI†) above a certain concentration, inferring a critical aggregation concentration (CAC). This aggregation affects the hydrodynamic volume leading to a decrease in *M*_n_ and *Đ* by GPC analysis compared to the theoretical values (Table 1). It must be noted, however, that the CAC was performed in H_2_O whilst the GPC was performed in DMF.

The zeta-potential values (*ζ*, Fig. 1E) of **P7**, **P8** and **P12** show a net negative charge due to the incorporation of GlcA monosaccharide into the glycopolymers, whilst the other glycopolymers (**P3–6**), which do not possess a charged monosaccharide, have a near-neutral zeta potential. Collating the CAC of the glycopolymers and the *ζ*-potential results, it can be proposed that repulsion between the negatively charged GlcA units resulted in an increase of the CAC by a factor of 10. The magnitude of the *ζ*-potential can also give information relative to the stability of the aggregates. The *ζ*-potential results show no significant difference between **P7** and **P8**, but **P12** exhibits a considerably larger negative zeta potential. By mimicking the alternation behaviour of the HA backbone, **P12** has an even charge distribution leading to the largest negative *ζ*, indicating the aggregate is more stable. For native HA, a much larger negative *ζ*-potential is observed compared to the glycopolymers **P7**, **P8** and **P12**. HA is a polyelectrolyte due to dissociation of the carboxylic acid of GlcA at physiological pH, which is repeated regularly across the HA chain at 1 nm intervals.^1^ Small-angle x-ray scattering (SAXS) on solutions of the glycopolymers also suggests differences on polymer's charge (Fig. 1F). The scattering pattern of natural HA exhibits a peak which is characteristic of a salt-free polyelectrolyte solution. This peak, often referred to as a ‘correlation peak’, represents an average distance between the charged domains in the solution (*i.e.* the HA chains).^37,38^ As it could be expected, due to the near-neutral *ζ*-potential values of the glycopolymers compared with HA, the scattering patterns of **P7**, **P8** and **P12** do not display a correlation peak of a polyelectrolyte.

A qualitative analysis of the scattering patterns of **P6**, **P7**, **P8** and **P12** could be fitted to the form factor of a semi-flexible chain (ESI,† eqn (1)).^39^ The best fits to the model are presented as solid lines in Fig. S11 (ESI†), and the best-fit parameters are given in Table S4 (ESI†). For different glycopolymers, the Kuhn length (∼50 Å) and the radius (∼15 Å) parameters are similar, suggesting that the flexibility of the chains does not change.

TEM (Fig. 1G) imaging on glycopolymer solutions above their CAC (Table S3, ESI†) shows the presence of aggregates. The glycopolymers displaying GlcA residues (**P7**, **P8** and **P12**) form smaller aggregates as observed by TEM and further analysed by ImageJ and DLS (Fig. S12, ESI†) showing a narrow range. Uranyl acetate was used as a stain for TEM imaging, which is known to interact with negatively charged groups, such as carboxylate groups. This might have contributed to the observed condensed structure of the more anionic glycopolymers (**P7**, **P8** and **P12**). By contrast, **P6** which bears no charge in the *ζ*-potential (Fig. 1E) forms larger aggregates with a wider range of sizes. This indicates that the balance between the hydrophobic and pi stacking attractions of the styrene within the backbone, and the charge repulsion of carboxylate groups on the GlcA residues, govern the size of the aggregates.

### CD44 binding

**Fig. 2:**
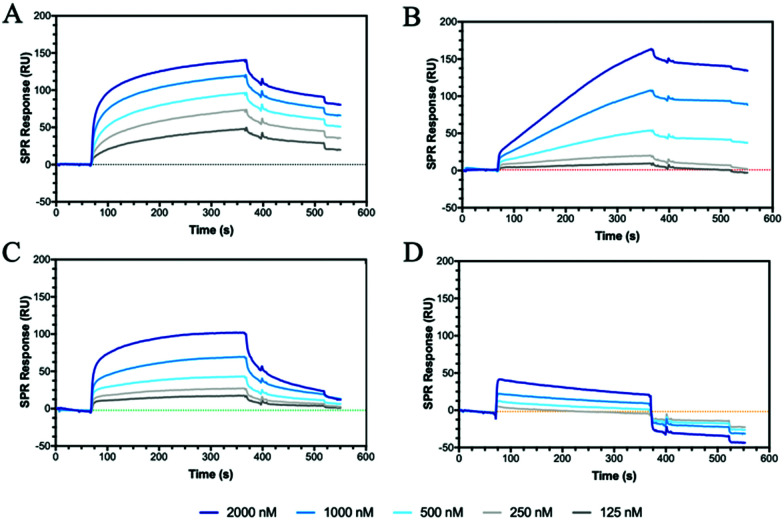
SPR sensorgrams for chips functionalised with CD44 at decreasing concentrations of analyte from 2000 to 125 nM of (A) **HA (5 kDa)**, (B) **P6**, (C) **P7** and (D) **P12**. The SPR experiments were conducted by first passing buffer flow for 60 seconds, followed by injection of the polymer solution for 300 seconds (association rate), and lastly buffer flow again for 200 seconds (dissociation rate).

CD44 is the major HA receptor which binds an octamer of HA in a groove or cleft on the side of the protein.^40^ To assess the ability of the glycopolymers to bind CD44, surface plasmon resonance (SPR) was used where recombinant human CD44 was coupled to a CM5 dextran chip through amine coupling (Fig. S13, ESI†). Initial SPR binding experiments were carried out with 20 kDa HA, but it was seen that the protein became saturated even at very low concentrations (Fig. S14, ESI†). By reducing the molecular weight from 20 to 5 kDa, an SPR sensorgram (Fig. 2A) was obtained showing concentration dependency, leading to a *K*_D_ of 0.15 μM, a value lower than previously reported by SPR (3.4–428 μM).^41,42^

The two glycopolymers **P6** (Fig. 2B) and **P7** (Fig. 2C) also show binding to CD44, with estimated affinities of 0.6 μM to 0.9 μM, respectively, although the value for **P6** has low confidence because no *R*_max_ could be reached and the sensorgrams suggest irreversible binding on the surface.

Control glycopolymers (**P3**, **P4** and **P5**) bearing glucose, mannose and galactose monosaccharides show no binding to CD44. For the statistical glycocopolymer (**P8**) and alternating copolymer (**P12**, Fig. 2D), no selective binding is observed despite them both possessing the HA monosaccharides. From these results, it is clear that CD44 shows selective binding to the monosaccharides found in HA, but when displayed in a specific configuration. The conformation of the CD44 binding cleft may enable enhanced interactions with the linear configuration of HA compared to the spherical aggregates of the glycopolymers which morphology has been shown to be critical for binding to other receptors.^43,44^ The binding of **P6** and **P7** is expected to follow a multivalent approach, whereby an increased number of localised monosaccharides allow for enhanced binding to receptors. In contrast, the disordered structures formed by **P8** and **P12**, displaying two monosaccharides, may prevent the multivalent effect.

### Peptide binding

**Fig. 3:**
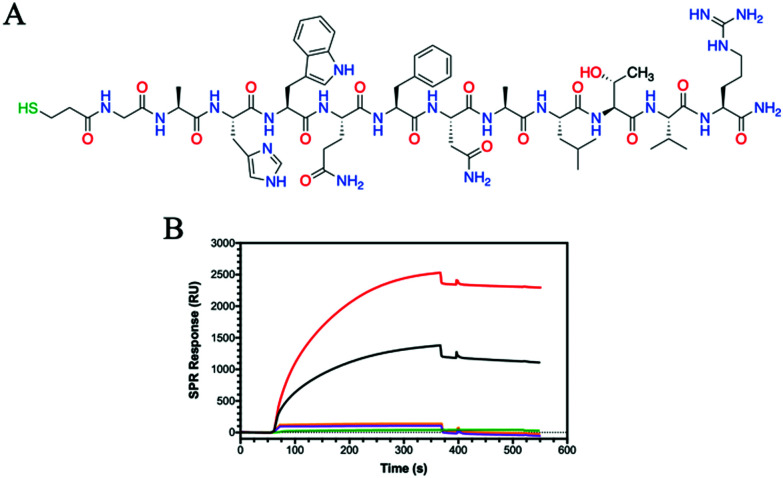
(A) Chemical structure of thiolated Pep-1 for immobilization on SPR gold chip. (B) SPR sensorgrams for binding to Pep-1 using **HA (5 kDa)** (black) and the glycopolymers of **P6** (red), **P7** (green), **P8** (purple) and **P12** (orange) at 2000 nM.

Having observed that some HA glycohomopolymers bind to CD44 with an affinity similar to HA, we also tested their ability to interact with HA-binding peptides. We have used a 12-mer peptide (GAHWQFNALTVR) named Pep-1, which was discovered by phage display and shown to bind HA with an affinity (*K*_D_) of 1.65 mM.^45^ Compared to large proteins, such as CD44, which require their complex tertiary structure for binding, Pep-1 is able to bind HA despite the random coil structure shown by circular dichroism (Fig. S17, ESI†). Peptides are easy to synthesize, and desired functionalities can be added during synthesis for facile immobilization on surfaces. To enable immobilization on gold surfaces, a thiol moiety was appended at the peptide N-terminus. The thiolated Pep-1 (Fig. 3A and Scheme S3, ESI†) was successfully synthesized and purified as confirmed by HPLC and ESI-MS analysis (Fig. S16, ESI†). The immobilisation of the peptide onto a gold surface was confirmed by contact angle (Fig. S18, ESI†) and SPR (Fig. S19, ESI†) analysis. The binding between a thiol and gold substrate is much faster compared to the attachment of the protein by amine coupling onto the cyclodextrin surface (Fig. S13, ESI†).

SPR results showed Pep-1 binding to 5 kDa HA with a *K*_D_ = 9.5 μM, a value similar to that originally determined.^45^ Comparing the binding of the glycopolymers to Pep-1 with 5 kDa HA, the binding of HA is surpassed by **P6** (Fig. 3B). **P6** contains only GlcNAc, which bears a hydrophobic *N*-acetyl group and shows a *K*_D_ of 68 nM, approximately 140 times more potent at binding to Pep-1 than HA. Alanine scanning studies on Pep-1 suggested the importance of hydrophobic residues for HA binding.^45^

Whilst Pep-1 possess a positive charge, due to the arginine situated at the C-terminus, no binding was observed with **P7** and the other GlcA bearing glycopolymers **P8** and **P12** (Fig. 3B and Fig. S20, ESI†). The SPR sensorgrams suggest that the hypothesis of hydrophobic interaction between HA and Pep-1 is still confirmed by this analytical technique. Similarly, the other glycopolymers **P3–P5** showed no binding to Pep-1 (Fig. S20, ESI†), indicating the specificity of Pep-1 towards HA and specifically the GlcNAc units.

### Lectin binding

HA interactions with toll-like receptors (TRLs), immune receptors that participate in the innate defence against bacterial infection, have been described in the literature.^46^ For example, HA oligosaccharides were shown to activate dendritic cells *via* TRL-4.^47^ Therefore, we have extended our binding studies to lectins, carbohydrate-binding proteins also involved in pathogen invasion and immune system activation.^48^ C-type lectins, such as DC-SIGN, MBL and SP-D, predominantly bind monosaccharides through the C3 and C4 hydroxides, often mediated by a metal ion such as calcium.^49^ However, as lectins are found as clusters on the cell surface, glycopolymers may be able to bind across multiple sites and produce an amplified lectin signal.^8^ Using a series of these C-type lectins and our SPR assay, HA showed no specific binding to DC-SIGN, MBL or SP-D, whilst the various glycopolymers display differing binding specificities for each lectin (Fig. S21–S23, ESI†).

**Fig. 4:**
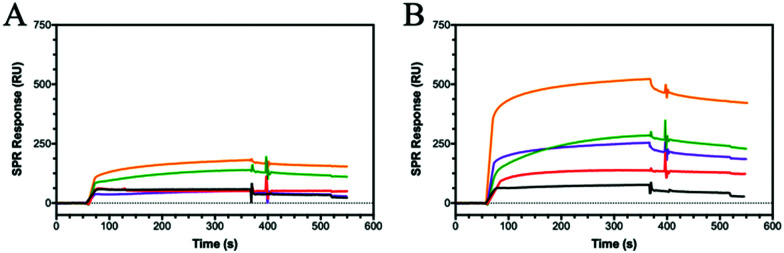
SPR sensorgrams showing (A) Dectin-1 and (B) DEC-205 binding to **HA (5 kDa)** (black) and the glycopolymers of **P6** (red), **P7** (green), **P8** (purple) and **P12** (orange) all at 2000 nM.

Dectin-1 (CD369)^50,51^ is a group V lectin found on the cell membrane surface and part of the natural killer cell receptor.^52^ Unlike DC-SIGN, MBL and SP-D, it binds saccharides without the requirement of a Ca^2+^ ion and, remarkably, binds β(1–3) glycans. The binding is enhanced for carbohydrates that also include β(1–6) bonds. HA possesses both β(1–4) linkages and, more importantly, a β(1–3) linkage suggesting that HA may bind to Dectin-1. SPR sensorgrams using Dectin-1 shows that HA does bind to the lectin (Fig. 4A). Our defined glycopolymer probes were used to investigate this further. The controls of Glc (**P3**), Man (**P4**) and Gal (**P5**) show no affinity for Dectin-1 (Fig. S24, ESI†), whereas the glycopolymers of HA did show binding. Of the two monosaccharides found in HA, a clear preference is seen for **P7** (Fig. 4A) compared to **P6**. When examining the crystal structure of the binding site within the lectin, several hydrophilic residues line the surface of the binding cleft, including histidine's and arginine's which can interact electrostatically with the carboxylic acid of GlcA, whilst other residues such as serine and aspartic acids can interact through hydrogen bonding. When comparing the *K*_D_ values of the GlcA-containing glycopolymers **P7**, **P8** and **P12**, the lowest *K*_D_ is seen for **P7** with a *K*_D_ of 2 pM (Fig. S24, ESI†) with a large component of this contributed by very slow dissociation rates showing that these interactions are very stable. The results may indicate Dectin-1 has specificity for negatively charge saccharides although this assumption requires additional investigations.

DEC-205 is a cell surface receptor in dendritic cells and has been shown to play a role in sensing cell-death.^53,54^ We tested the binding of glycopolymers to DEC-205 (Fig. 4B) using SPR and found that HA bound rather poorly, similar to Dectin-1 (Fig. 4A). Whilst DEC-205 is thought to be a C-type lectin, it appears to share some binding similarities with Dectin-1 and CD44 based on the SPR data. The crystal structure for DEC-205 has not been reported, but the literature suggests that DEC-205 is a Man-type lectin.^55^ When studying the binding patterns, no specific binding is seen for **P4** or **P5**, but **P3** showed binding, inferring that this lectin is actually a glucose-binding lectin. This hypothesis is extended by the binding of **P6** and **P7**, which are glucose-based epimers. When comparing the binding affinities of **P6** and **P7**, an order of magnitude difference is observed with *K*_D_ of 100 nM and 17 nM, respectively. These trends are similar to Dectin-1, suggesting a similar binding model. The binding affinities are enhanced by the formation of a statistical copolymer containing both monosaccharides giving 2 orders of magnitude decrease in *K*_D_ for **P8** (*K*_D_ = 910 pM) with a further order of magnitude decrease for **P12** (*K*_D_ of 13 pM). This implies the distribution of GlcA is critical for the binding of HA to DEC-205. The results suggest that glycopolymers can be designed to enhance interactions with specific lectins by appropriate spacing of the carboxylic acid and GlcNAc units. The binding results for all the proteins and peptide are summarised in Table S5 (ESI†).

### Hyaluronidase binding and inhibition

Hyaluronidase (HAase), the enzyme that catalyses the hydrolysis of HA by cleavage of β(1–4) glycosidic linkages, plays an important role in HA turnover in the human body.^56^ High levels of HAase are hallmarks in many types of cancer.^57,58^ Thus, modulating their activity through inhibitors is critical for normal homeostasis and for designing cancer therapies. The potential of the HA mimetic glycopolymers as HAase inhibitors was evaluated by first assessing their interaction with HAase by SPR. Heparin, a well-known non-competitive inhibitor of HAase, which does not bind to the enzyme catalytic site, was used as a control.^59,60^ However, the heparin inhibition effects on HAase activity are achieved for heparin concentrations higher than the ones present at physiological levels.^61^

**Fig. 5:**
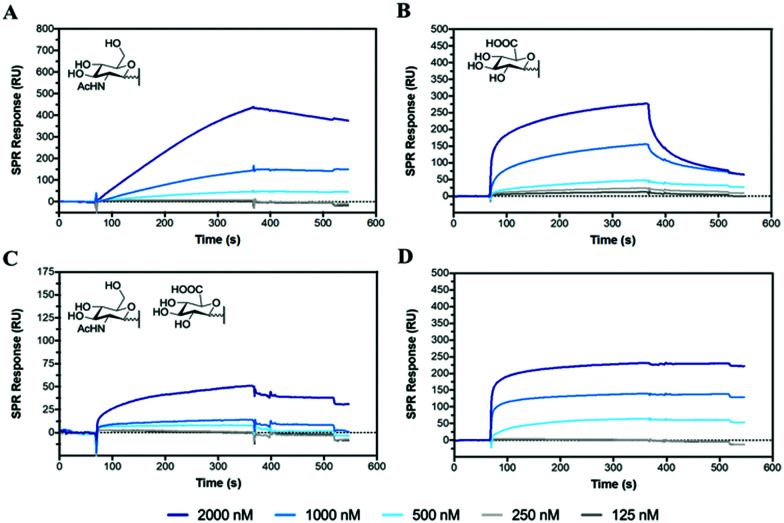
SPR sensorgrams for chips functionalised with HAase challenged with concentrations of analyte from 2000 to 125 nM of (A) **P6**, (B) **P7**, (C) **P8** and (D) **heparin**.

The glycopolymers bearing monosaccharides of GlcNAc (**P6**) and GlcA (**P7**) bound to HAase with similar binding profiles as observed with CD44 (Fig. 2), with the rapid association for **P7** and slower, but tighter association for **P6** (Fig. 5A and B). Additionally, the statistical copolymer (**P8**, Fig. 5C) displayed a weak interaction with HAase at the highest concentration. Heparin (Fig. 5D) associated rapidly and showed unmeasurably slow dissociation. The result infers that electrostatic interactions^62^ play an important role in the rate of association to HAase since heparin, **P7** and **P8**, all possessing negative charges, associate faster than the neutral **P6** glycopolymer (Table S5, ESI†). The other glycopolymers (**P3**, **P4**, **P5** and **P12**) showed no binding to HAase (Fig. S26, ESI†).

**Fig. 6:**
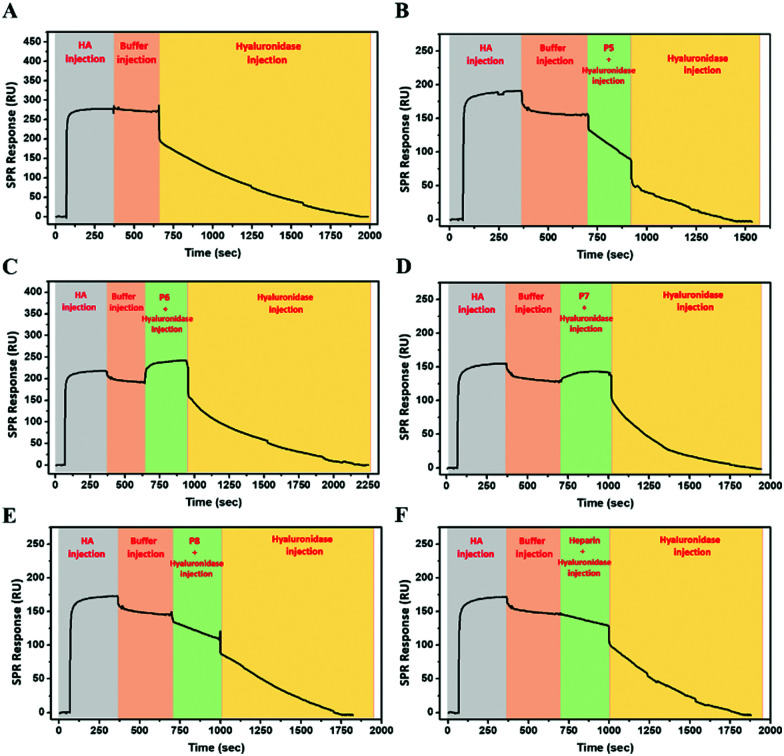
SPR measurements showing the inhibitory activity of the glycopolymers against hyaluronidase on the degradation of HA bound to the immobilized CD44 receptor; (A) without polymer, (B) **P5**, (C) **P6**, (D) **P7**, (E) **P8**, and (F) **heparin**.

To investigate the potential of glycopolymers as inhibitors of HAase activity, we have used SPR real-time interaction measurement to monitor binding, inhibition and degradation in a single run. CD44 was firstly bound to the chip surface to immobilize HA. HAase was then incubated with appropriate glycopolymers or heparin before this mixture was injected over the SPR chip surface. As seen in Fig. 6A, when HAase without inhibitor was injected into the system, there was a rapid and dramatic reduction in SPR units showing the total mass decreased on the chip surface because of the degradation and release of HA.

**P5** was used as control glycopolymer as no binding was observed between **P5** and HAase (Fig. S26, ESI†). HA degradation by HAase proceeds in the presence of **P5** (Fig. 6B), as anticipated. Mixing of HAase with **P6** or **P7** resulted in additional mass on the chip suggesting that the HAase could bind HA, but these glycopolymer–HAase complexes may cause the inactivity or inhibition of the HAase towards HA degradation (Fig. 6C and D). Subsequent injection of HAase alone led to resumption of loss of mass from the chip in both cases. **P8** and heparin (Fig. 6E and F) displayed retardation in the rates of HA degradation and no rise in mass on the chip surface, which suggests that these polymers block the ability for HA to bind to the HAase binding site as part of their mechanism of inhibition.

In summary, even though the SPR results indicate that **P6** and **P7** displayed stronger inhibition of HAase activity than heparin, these preliminary results require further investigation using different techniques to assess the degree and mechanism of HAase inhibition by these HA glycopolymers.

The diversity of the glycopolymers tested in this study offers the possibility to probe, in isolation or combination, the effect of hydrophobic interactions (GlcNAc in **P6**), electrostatic interactions (GlcA in **P7**), or both (**P8** and **P12**), on the binding to protein receptors or the difference between the epimers of Glc-based (**P3**, **P6**, **P7**, **P8** & **P12**), Man-based (**P4**) and Gal-based (**P5**).

### Cytotoxicity of the HA glycopolymers

To extend the utility of the HA glycopolymers as chemical probes in cell studies, their cytotoxicity was evaluated on relevant cells, such as LuC4 cells (cell line derived from oral and skin carcinomas) which express CD44.^63^ The cell viability results (Fig. S27A, ESI†) showed that the glycopolymers were non-toxic to cells. No statistically significant difference was seen between the control and **P6**, **P7** and **P12**. Incubation with **P8** resulted in a statistically significant decrease in the normalised fluorescent output of the calcein-AM stain. However, upon analysis of fluorescence of dead cells stained with ethidium homodimer-1 (Fig. S27B, ESI†), there was no significant difference seen between each of the glycopolymers. The results indicate that the glycopolymers have low cytotoxicity up to a concentration of 100 μg mL^−1^.

## Conclusions

Using controlled polymerization combined with CuAAC click reaction, HA-based glycopolymers were successfully produced, including glycohomopolymers and a statistical polymer based on HA monosaccharides. Through a selective and orthogonal reaction, a fully alternating HA glycopolymer was produced by selective grafting of monosaccharides to each individual monomer of a modified styrene-maleic anhydride polymer. The glycopolymers were used as probes to identify monosaccharides involved in the binding interactions with known proteins (CD44, HAase) and peptides. The HA glycopolymer probes were further analysed for binding to both classical and non-classical C-type lectins, confirming that the latter bind to HA. The backbone stiffness and monosaccharide composition/presentation were shown to play an important role in the glycopolymer conformation and in the interaction with HA- and carbohydrate-binding proteins. Moreover, our studies reveal the value of HA glycopolymers as potential synthetic inhibitors of HA-binding proteins.

## Author contributions

D. W. P. C. undertook the glycopolymer and peptide synthesis and their chemical characterization (FTIR, NMR, GPC, DLS, MALDI, HPLC, zeta potential, contact angle) and co-wrote the manuscript; G. Y. designed and performed the SPR for the hyaluronidase experiments, analysed the data and co-wrote the manuscript; Y. Y. collaborated in the hyaluronidase studies; G. O. and R. B. performed SAXS measurements, analysed the data and commented the manuscript; Y. S. performed TEM imaging; C. O’M. conducted the cell cytotoxicity studies; A. M., V. U. and R. N. performed SPR experiments, analysed the data and commented the manuscript; C. R. B. supervised the polymer synthesis work, binding studies with lectins and commented the results; H. S. A. conceived the idea and supervised the peptide synthesis work and co-wrote the manuscript. All authors read and approved the manuscript.

## Conflicts of interest

There are no conflicts to declare.

## Supplementary Material

CB-002-D0CB00223B-s001
