# Supplementary material for: Hyaluronan (HA)-inspired glycopolymers as molecular tools for studying HA functions
Source: RSC Chem Biol. 2021 Jan 28;2(2):568–76. doi: 10.1039/d0cb00223b (PMC8341579; doi:10.1039/d0cb00223b)
Supplement: CB-002-D0CB00223B-s001 [file CB-002-D0CB00223B-s001.pdf]

Electronic Supplementary Material (ESI) for RSC Chemical Biology.
This journal is © The Royal Society of Chemistry 2021

Electronic Supplementary Information

Hyaluronan (HA)-inspired neoglycopolymers as enabling molecular tools for studies of HA functions

Dominic W. P. Collis[1], Gokhan Yilmaz[1,5], Yichen Yuan[1], Alessandra Monaco[1,5], Guy Ochbaum[2], Yejiao Shi[1], Clare O'Malley[1,3], Veselina Uzunova[4], Richard Napier[4], Ronit Bitton[2], C. Remzi Becer[1,5], Helena S. Azevedo[1,3]

[1]School of Engineering and Materials Science, Queen Mary University of London, London E1 4NS, UK

[2] Department of Chemical Engineering and the Ilza Katz, Institute for Nanoscale Science & Technology, Ben-Gurion University of the Negev, Beer-Sheva 84105, Israel

[3] Institute of Bioengineering, Queen Mary University of London, London E1 4NS, UK

[4] School of Life Sciences, University of Warwick, CV4 7AL, UK

[5] Department of Chemistry, University of Warwick, CV4 7AL, UK

Materials

All chemicals were used without further purification unless stated otherwise.

For the glycopolymers: Vinylbenzyl chloride, N-acetyl-D-glucosamine, D-Glucuronic Acid, sodium azide, propargyl alcohol, propargyl acrylate, D-(+)-mannose, D-(+)-galactose, D-(+)-glucose, sodium methoxide, maleimide, dichloromethane, methanol, acetic acid, iodine, sulphuric acid, ethyl acetate, sodium acetate, boron trifluoride-diethyl etherate, dimethyl formaldehyde, iodomethane, 2-bromoethanol, tetrabutylammonium bromide, diethyl ether, dimethyl sulfoxide, triphenylphosphine, tetrahydrofuran, diisopropyl azodicarboxylate, 3-(trimethylsilyl)propargyl alcohol, hexane, and copper bromide were purchased from Sigma Aldrich. Sodium thiosulfate, magnesium sulfate, sodium chloride, sodium bicarbonate, silica, potassium carbonate and aluminium oxide were purchased from VWR. Before use in the polymerisations, all monomers were passed through basic Al2O3. Dialysis tubing with 1 kDa MWCO was purchased from Spectrum Laboratories. All proteins (CD-44, DC-SIGN, MBL, SP-D, DEC-205, Dectin-1) were purchased from R&D Systems. The CM5 sensor chip and the amine coupling kit for the immobilization of the proteins on the chip surface were purchased from GE Healthcare Life Sciences.

For the peptides: Fmoc-amino acids and 4-methylbenzhydrylamine (MBHA) rink amide resin were purchased from Novabiochem. 1-hydroxybenzotriazole hydrate (HOBt), N,N'-diisopropyl carbodiimide (DIC), piperidine, dimethylformamide (DMF), dichloromethane (DCM), trifluoroacetic acid (TFA), anisole, thioanisole, 1,2-ethanedithiol (EDT) triisopropyl silane (TIS), diethyl ether, acetonitrile (ACN), acetic anhydride (Ac2O), mercapto propionic acid, 4-methoxytriphenylmethyl chloride (MMT) and the Kaiser test kit were acquired from Sigma-Aldrich and were used without further purification.

Heparin sodium salt from porcine intestinal mucosa (Mw ~15 kDa, ≥180 USP units/mg) and hyaluronidase from bovine testes (Type IV-S, lyophilized powder (essentially salt-free), Mw ~55 kDa, 750-3000 units/mg solid) were purchased from Sigma-Aldrich.

Analytical methods

Proton nuclear resonance (^{1}H NMR) and carbon nuclear resonance (^{13}C) spectra were recorded on a Bruker AV-III spectrometer at 400 MHz using $CDCl_3$, DMSO-d_6, Acetone-d_6 or D_2O as a solvent.

Gel Permeation Chromatography (GPC) was performed on an Agilent 1260 infinity system operating in DMF with 5 mM NH_4BF_4 and equipped with refractive index detector and variable wavelength detector, 2 PLgel 5 µm mixed-C columns (300 × 7.5 mm), a PLgel 5 mm guard column (50 × 7.5 mm), a differential refractive index (DRI) and a variable wavelength detector (VWD). The system was eluted with DMF at a flow rate of 1 mL min^{-1} and the DRI was calibrated with linear narrow polystyrene standards. All the samples for GPC were previously filtered through a 0.22 µm filter.

Infrared spectra were collected on a Tensor 27 (Bruker) FTIR spectrometer cooled by $N_{2\ (l)}$ coupled with a platinum attenuated total reflectance (ATR) for both solid and liquid samples.

The polymers mass was measured on a Bruker Daltonics AuToFlex MALDI-ToF mass spectrometer, with a nitrogen laser at 337 nm and equipped with positive ion ToF detection. A 9:1:1 solution of:

matrix (30 mg/mL), potassium trifluoroacetate (10 mg/mL) and the sample (10 mg/mL) dissolved in THF, with 2 µL being deposited onto the stainless-steel target plate. Spectra were recorded in reflectron mode with the laser set at 20-30% power and masses determined against 200-6000 Da calibration.

ζ-potential of glycopolymers was measured on a Nano-ZS Zetasizer (Malvern) at 100 µM at pH 7.4.

Small angle x-ray scattering patterns of glycopolymer solutions (200 µM) were obtained with a SAXSLAB GANESHA 300-XL. CuK_{α} radiation was generated by a Genix 3D Cu-source with an integrated monochromator, 3-pinhole collimation and a two-dimensional Pilatus 300K detector. The scattering intensity q was recorded at intervals of $0.012 < q < 0.3$ Å^{-1} (corresponding to lengths of 10-800 Å). Measurements were performed under vacuum at the ambient temperature. The scattering curves were corrected for counting time and sample absorption. The solution under study was sealed in thin-walled quartz capillaries about 1.5 mm in diameter and 0.01 mm wall thickness. The scattering spectra of the solvent were subtracted from the corresponding solution data using the Irena package for analysis of small-angle scattering data[1] Data analysis was based on fitting the scattering curve to an appropriate model by software provided by NIST (NIST SANS analysis version 7.0 on IGOR).[2]

Model fitting of the small-angle scattering pattern

The form factor of a semiflexible chain with excluded volume effects is expressed by[3, 4]:

$$P(q,b,L,R_{cs}) = P_{exv}(q,b,L) + C(L/b)\frac{b}{15L}\left[4+\frac{7}{u}-\left(11+\frac{7}{u}\right)e^{-u}\right]\cdot\left[2\frac{J_1(q,R_{cs})}{q,R_{cs}}\right] \qquad \text{(eq. 1)}$$

$$P_{exv}(q,b,L) = \left[1-w(qR_g)\right]\cdot P_{Debye}(q.b.L) + w(qR_g)\left[C_1(qR_g)^{-1/v}C_2(qR_g)^{-2/v}C_3(qR_g)^{-3/v}\right] \qquad \text{(eq. 2)}$$

This describes a chain comprised of a series of locally stiff segments by three parameters b - Kuhn's length, a measure of the chain flexibility, L - the chain's contour length, and R - radius of the chain's cross-section.

The mass of the peptide was determined using an LC-MS Agilent '1100 LC/MSD Trap' in methanol.

Circular dichroism (CD) was used to measure the secondary structure of the peptide on a Chirascan CD spectrometer (Applied Photophysics) from 190 to 300 nm at 25 °C in H_2O at 110 µM in a 3 mm quartz cuvette, with a blank run in pure water.

TEM analysis was carried out on glycopolymer solutions at 1 mM at pH 7 after being aged for 48 hours. The glycopolymer solutions were loaded onto the carbon film-coated copper grids (400 mesh, Agar Scientific, UK) and negatively stained by 2 wt% uranyl acetate (Agar Scientific, UK). The excess staining solution on the grids was removed with filter paper and the grids were allowed to dry at room temperature for at least 3 hours. Bright-field TEM imaging was performed on a JEOL 1230 TEM operated at an acceleration voltage of 100 kV and the TEM images were recorded by an SIS Megaview III wide-angle CCD camera

Dynamic Light Scattering (DLS) on the glycopolymers was performed in a Nano-ZS Zetasizer (Malvern Instruments). Glycopolymer solutions were prepared above the CAC point at 1 mM at pH 7 and allowed to stand for 2 days to ensure s formation of aggregates. The sample was diluted 10-fold and

passed through a 0.2 µm filter. The cell and the DLS machine were cleaned using compressed air to ensure removal of large dust particles, before reading.

Experimental Methods

Cell Viability

Cell viability was assessed using the LIVE/DEAD™ viability/cytotoxicity kit for mammalian cells (Invitrogen) and following the manufacturer's instructions. Briefly, LuC4 cells (expressed for a human oral squamous cell carcinoma cell line) were seeded at 5,000 cells per well into a 96 well plate and grown in a humidified atmosphere at 37 °C, 5% CO_2 for 48 hours. Cells were treated with glycopolymers at a range of concentrations, 0.1, 1, 10, 100, µg/mL, and incubated for 24 hours. Media was removed, and live/dead dyes (green-fluorescent calcein-AM/red-fluorescent ethidium homodimer-1) were added in PBS and incubated for 1 hour. Fluorescence was read using Synergy HT (BioTek) at 530 nm for calcein-AM (live cells) and at 645 nm for ethidium homodimer-1 (dead cells). The number of live and dead cells was calculated as a percentage of the fluorescent output compared to a control containing no glycopolymer for the live control and methanol fixed cells for the dead assay.

Self-Assembled Monolayers (SAMs) of HA-binding peptide (Pep-1)

The formation of the Pep-1 SAMs was performed on gold-coated coverslips, cleaned using basic piranha mixture before use. The clean gold surfaces were placed in an ethanoic solution of thiolated Pep-1 (1% w/v) for 24 hours at 37 ºC, after which the surface was washed with water and dried under a nitrogen stream. The contact angle was measured using a Drop Shape Analyzer – DSA100 (Kurss). The incubation process was repeated in an aqueous solution of 1.5 MDa HA (0.5 % w/v). The contact angle was again measured and compared to a control of a bare gold surface which had been treated in a similar way but with no peptide or HA in the incubation solutions.

Surface Plasmon Resonance (SPR)

A BIAcore 2000 system SPR (Cytiva) was used for interaction analysis. The CD44 protein (Recombinant Human CD44 Fc Chimera Protein, 48.6 kDa, 0.025 mg/ml) in 10 mM sodium acetate buffer solution at pH 5.5 was immobilized via a standard amino coupling protocol onto a CM5 sensor chip that was activated by flowing a 1:1 mixture of 0.1 M N-hydroxysuccinimide and 0.1 M N-ethyl-N'-(dimethylaminopropyl) carbodiimide over the chip for 6 min at 25 °C at a flow rate of 20 µL/min. Subsequently, active channels including 1 (blank) were blocked with a solution of ethanolamine (1 M, pH 8.5) for 10 min at 5 µL/min. Polymer solutions were prepared at varying concentrations (2000 nM-125 nM) in PBS buffer plus 0.005% Tween20 at pH 7.4. Sensorgrams for each glycopolymer concentration were recorded with a 300 seconds injection of polymer solution (association) followed by 150 seconds of buffer alone (dissociation). Regeneration of the sensor chip surfaces was performed

using 100 mM glycine HCl at pH 2.0. Kinetic data were evaluated using a single set of sites (1:1 Langmuir Binding) model and also a bivalent model in BIAevaluation 3.1 software.

All lectins (Recombinant Human Proteins, 0.025 mg/ml) were immobilized as described above and the system was equilibrated with filtered HEPES buffer (10 mM HEPES pH 7.4, 150 mM NaCl, 5 mM $CaCl_2$, 0.01% P20 surfactant solution). For the immobilization of Pep-1, a gold surface without any modification was used and thiolated peptide (1 mg/ml) was run at a flow rate of 2 µL/min for 15 min at 25 °C to provide high attachment of peptide on the gold surface. Regeneration of the sensor chip surfaces was performed using 10 mM HEPES pH 7.4, 150 mM NaCl, 10 mM EDTA, 0.01% P20 surfactant solution for the lectins. For regeneration of peptides immobilized surface, 50 mM NaOH solution was used. Hyaluronidase (0.025 mg/ml) was immobilized as described above for lectins. A 1-min pulse of a mixture of 50 mM NaOH and 1 M NaCl was used to regenerate the surface after each binding cycle.

For the HAase inhibition experiments, CD44 protein (0.025 mg/ml) was first immobilized on the chip surface and then HA (20 kDa) solution at 2000 nM was added to form a complex between CD44 and HA. Subsequently, hyaluronidase HA solutions previously incubated with various glycopolymers (2000 nM and 30 min incubation) were injected for 5 min at 10 µL/min. Following this, a flow of HAase solution at 2000 nM was passed across the chip without inhibitors. Regeneration of the sensor chip surfaces was performed using 100 mM glycine HCl at pH 2.0.

Glycopolymer solutions were prepared (2000 nM-125 nM) in the appropriate PBS or HEPES buffer.

Synthetic Procedures

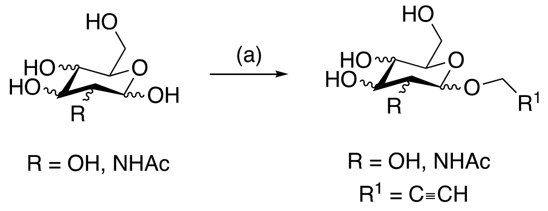

Scheme S1. Direct glycosidation of monosaccharides. Reagents and condition: (a) propargyl alcohol (neat), H_2SO_4, 65 ºC

General procedure for the synthesis of Prop-2-ynyl monosaccharides

The synthesis has been modified from previously reported method[5]. The example is given for Prop-2-ynl 2-acetamido-2-deoxy-D-glucopyranoside**.** Briefly, N-acetyl-glucosamine (10.01 g, 45.3 mmol) was suspended in propargyl alcohol (25 mL, excess) which was heated to 65 ºC. H_2SO_4.Silica (1.30 g, 30 mg per mmol) was added and the reaction was stirred for 16 hours. The reaction mixture was added to a silica column with the propargyl alcohol flushed out with DCM and then the product flushed out with methanol. The product was reduced under negative pressure with the resulting oil being suspended in water and lyophilized to produce a yellow powder.

Prop-2-ynl-D-glucopyranoside

^{1}H NMR (400 MHz, $CDCl_3$) δ: 5.11 (d, 0.4H, 3.8 Hz H-1), 4.67 (d, 0.6 H,12 Hz, 1H, H-1), 4.50 (t, 3.0 Hz, H-2), 4.36 (1H, q, 2.3 Hz CH≡CH), 4.25 (d, 0.6H, 2.4 Hz) 3.96–3.69 (3H, m, H-6, H-5) 3.55-3.29 (2H, m H-4, H-3), 2.93 (1H, dt, 11 Hz, 2.4 Hz, CH≡CH).

Prop-2-ynl-D-mannopyranoside

^{1}H NMR (400 MHz, $CDCl_3$) δ: 5.05 (d, 0.4H, 1.6 Hz H-1), 4.67 (d, 0.6 H, 12 Hz, 1H, H-1), 4.50 (t, 3.0 Hz, H-2), 4.32 (1.4H, dd, 6.1 Hz, 2.4 Hz CH≡CH), 4.25 (d, 0.6H, 2.4 Hz, CH≡CH) 3.96–3.68 (5H, m, H-6, H-5, H-4, H-3), 2.94 (0.75 H, t, 2.4 Hz, CH≡CH) and 2.86 (0.75 H, t, 2.4 Hz, CH≡CH).

Prop-2-ynl-D-galactucopyranoside

^{1}H NMR (400 MHz, $CDCl_3$) δ: 5.17 (dd, 0.75H, 15.6 Hz, 2.8 Hz H-1), 4.59 (d, 0.25 H,12 Hz, 1H, H-1), 4.36 (1.4H, dd, 2.4 Hz CH_2≡CH), 4.25 (d, 0.6H, 2.4 Hz, CH≡CH) 4.12–3.67 (5H, m, H-6, H-5, H-4, H-3), 2.93 (0.75 H, t, 2.4 Hz, CH≡CH) and 2.85 (0.75 H, t, 2.4 Hz, C_2≡CH).

Prop-2-ynl 2-acetamido-2-deoxy-D-glucopyranoside

^{1}H NMR (400 MHz, $CDCl_3$) δ: 5.10 (d, 0.4H, 4 Hz H-1), 4.77 (d, 0.6 H,12 Hz, 1H, H-1), 4.36 (1H, q, 14.0 Hz, 2 Hz CH≡CH), 3.98 (1H, t, H-2), 3.74 – 3.84 (3H, m, H-6, H-5), 3.50-3.63 (2H, m, H-4, H-3), 2.96 (1H, dt, 11 Hz, 2.4 Hz, CH≡CH), 2.09 (3H, s, NH(O)CH_3).

FT-IR (ATR): 3274 (br, OH), 2931 (w, C-H), 2118 (w, C≡C), 1650 (s, C=O).14, 1556, 1438, 1417, 1387, 1316, 1254 (w, C-O), 1099, 1079, 1026, 950, 890, 662, 525

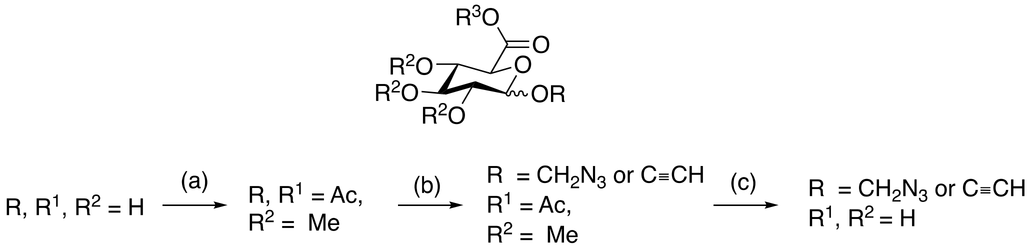

Scheme S2. Indirect glycosidation of GlcA. Reagents and condition: (a) (i) Ac_2O, H_2SO_4 (ii) MeI, $NaHCO_3$ (b) alcohol donor, $BF_3.OEt_2$, DCM, 0 ºC (c) MeONa, MeOH

1,2,3,4-tetra-O-acetyl-α-D-glucopyranuronate

The synthesis has been modified from previously reported method[6]. Briefly, glucuronic acid (5.14 g) was added to a stirring solution of Ac_2O (25 mL, 264 mmol) and H_2SO_4 (5 drops) which was then heated to 65 ºC. Once the added sugar was dissolved, more glucuronic acid (5.09 g) was added (Total glucuronic acid; 10.23 g, 52.7 mmol). The solution was stirred for 1 hour and then cooled to 25 ºC. H_2O (75 mL) was added and then the product was extracted into DCM (3 x 50 mL). The organic layers were combined, dried over $MgSO_4$ and reduced under negative pressure and azeotrope against toluene. The product was used without further purification.

^{1}H NMR (400 MHz, $CDCl_3$) δ: 6.43 (bs, 1H COOH), 5.78 (d, 7.5 Hz, 1H, H-1), 5.29 (m, 2H, H-2, H-5), 5.12 (m, 1H, H-3), 4.24 (m, 2H, H-4), 2.10, 2.03, 2.02, 2.01 (4s, 12H, OAc).

FT-IR (ATR): 3303 (br, O-H), 2950 (w, C-H), 1756 (s, C=O), 1726 (s, C=O) 1392, 1369, 1510, 1361, 1205 (C-O), 1122, 1045, 1005, 892, 657, 597, 565, 495

Methyl 1,2,3,4-tetra-O-acetyl-α-D-glucopyranuronate

The crude 1,2,3,4-tetra-O-acetyl-α-D-glucopyranuronate was dissolved in DMF (25 mL) and reacted with $NaHCO_3$ (8.9 g, 106 mmol) followed by the addition of MeI (8.2 mL, 132 mmol). The reaction was stirred for 16 hours at 25 ºC. The reaction was quenched with H_2O (200 mL) and the product extracted into EtOAc (3 x 200 mL). The organic layers were combined, dried over $MgSO_4$ and concentrated under negative pressure. The resulting oil was taken up in acetone and precipitated in water. The precipitate was filtered and dried under vacuum to give a white powder (12.74 g, 33.9 mmol, 64 %).

^1H NMR (400 MHz, $CDCl_3$) δ: 5.76 (d, 7.7 Hz, 1H, H-1), 5.31 (t, 10.0 Hz, 1H, H-3), 5.25 (t, 9.4 Hz, H-4), 5.14 (t, 8.9 Hz, 1H, H-2), 4.18 (d, 9.5 Hz, 1H, H-5), 3.74 (s, 3H, OCH_3), 2.11 (s, 3H, CH_3), 2.03 (s, 6H, 2 x CH_3), 2.02 (s, 3H, CH_3).

FT-IR (ATR): 2948 (w, C-H), 1749 (s, C=O), 1436, 1369, 1209 (s, C-O), 1051, 1037, 889, 597, 505

2-Azidoethanol

The synthesis was based in a previously reported method[7]. Briefly, 2-bromoethanol (10 g, 80 mmol) was suspended in H_2O (20 mL) followed by the addition of NaN_3 (9.53 g, 144 mmol) and NBu_4Br (590 mg). The reaction was stirred for 16 hours at 80 ºC. The reaction was cooled and extracted into Et_2O (10 x 50 mL). The organic layers were combined and reduced to give the product as a clear oil.

^1H NMR (400 MHz, $CDCl_3$) δ: 3.73 (t, 4.5 Hz, 2H, CH_2OH), 3.38 (t, 4.5 Hz, 2H, CH_2N_3).

FT-IR (ATR): 3373 (br, O-H), 2935 (w, C-H), 2098 (s, N_3), 1441, 1347 1283 (w, C-O), 1060, 977, 877, 828, 631, 554, 503

General procedure for the glycosidation by Lewis acid

Methyl 1,2,3,4-tetra-O-acetyl-α-D-glucopyranuronate (1 equiv) and propargyl alcohol (4 equiv) were suspended in anhydrous DCM (25 mL). The reaction was cooled to 0 ºC at which point $BF_3.OEt_2$ (2 equiv) was added. The reaction was stirred at 0 ºC for 15 minutes and then allowed to warm to 25 ºC and stirred for 16 hours. The reaction was diluted with DCM and then washed with water (2 x 50 mL) and brine (50 mL). The organic layers were combined, dried over $MgSO_4$ and reduced under negative pressure. The oil was purified by flash chromatography, EtOAc: hexane (1:1).

Methyl 2,3,4-tri-O-acetyl-1-propargyl-α-D-glucuronate

Methyl 1,2,3,4-tetra-O-acetyl-α-D-glucopyranuronate (5.06 g, 13.4 mmol) and propargyl alcohol (3.2 mL, 54.8 mmol) were suspended in anhydrous DCM (25 mL). The reaction was cooled to 0 ºC at which point $BF_3.OEt_2$ (3.3 mL, 25.3 mmol) was added producing an off white solid (2.94 g, 7.90 mmol, 59%).

^{1}H NMR (400 MHz, $CDCl_3$) δ: 5.49 (t, 10.1 Hz, 1H, H2), 5.21 (q, 9.4 Hz, 2H H3, H4), 5.09 (dd, 8.4 Hz, 3.7 Hz, 1H, H5), 4.63 (d, 7.7 Hz, 0.4 Hz, **H**1), 4.38 (d, 10.2 Hz, 0.4 Hz, H1), 4.21 (t, 5.1 Hz, 2H, C**H_2**N_3), 3.73 (s, 3H, OC**H_3**), 3.45 (t, 5.2 Hz, 2H, OC**H_2**), 2.48 (m, 1H, C**H**) 2.02 (s, 3H, C**H_3**), 2.01 (s, 3H, C**H_3**), 1.98 (s, 3H, C**H_3**).

FT-IR (ATR): 3278 (w, C-H), 1743 (s, C=O), 1437, 1369, 1212, 938, 897, 651, 599, 529

Methyl (2-azidoethyl 2,3,4-tri-O-acetyl-β-D-glucopyranosid)uronate

Methyl 1,2,3,4-tetra-O-acetyl-α-D-glucopyranuronate (5.03 g, 13.4 mmol) and 2-azidoethanol (2.6 mL, 52.6 mmol) were suspended in anhydrous DCM (25 mL). The reaction was cooled to 0 ºC at which point $BF_3.OEt_2$ (3.3 mL, 25.3 mmol) was added.

^{1}H NMR (400 MHz, $CDCl_3$) δ: 5.49 (t, 10.1 Hz, 1H, H2), 5.21 (q, 9.4 Hz, 2H H3, H4), 5.09 (dd, 8.4 Hz, 3.7 Hz, 1H, H5), 4.63 (d, 7.7 Hz, 0.4 Hz, **H**1), 4.38 (d, 10.2 Hz, 0.4 Hz, H1), 4.21 (t, 5.1 Hz, 2H, C**H_2**N_3), 3.73 (s, 3H, OC**H_3**), 3.45 (t, 5.2 Hz, 2H, OC**H_2**) 2.02 (s, 3H, C**H_3**), 2.01 (s, 3H, C**H_3**), 1.98 (s, 3H, C**H_3**).

FT-IR (ATR): 2957 (w, C-H), 2106 (s, N_3), 1743 (s, C=O), 1438, 1370, 1209, 1038, 936, 893, 599, 508

1-(3-(triisopropylsilyl)prop-2-ynyl)-1H-pyrrole-2,5-dione

The synthesis has been modified from previously reported method[8]. Briefly, triphenylphosphine (8.1 g, 30.9 mmol) was dissolved in THF (80 mL) and cooled to -78 °C under an inert atmosphere. DIAD (6.1 mL, 30.9 mmol) was added dropwise over 5 minutes followed by 3- (trimethylsilyl)prop-2-yn-1-ol (4.1 mL, 28.1 mmol) dropwise over 5 minutes. The maleimide (3 g, 31.6 mmol) was suspended in THF (100 mL) and added dropwise. The resulting foam was stirred for 10 minutes at -78 °C and then 24 hours at 25 ºC. The solvent was removed in vacuum and the residue purified twice by column chromatography: hexane: EtOAc (1:1) followed by hexane: EtOAc (2:1). The fractions were collected reduction under vacuum to give 1-(3-(trimethylsilyl)prop-2-ynyl)-1H-pyrrole-2,5-dione as a yellow oil.

^{1}H NMR (400 MHz, $CDCl_3$) δ: 6.74 (s, 2H, HC=CH), 4.28 (s, 2H, CH_2), 0.12 (s, 9H, $Si(CH_3)_3$.

FT-IR (ATR): 2961 (w, C-H), 2182 (w, C≡C), 1713 (s, C=O), 1425, 1401, 1344, 1249, 1151, 1041, 1007, 826, 759, 694, 654, 509.

General procedure for polymerisations

The synthesis has been modified from previously reported method[9]. The polymers were synthesised by RAFT polymerisation, using BDTMP as the RAFT agent and V601 as the initiator. Briefly, all the components were combined and purged in a Schlenk tube under nitrogen flow for 30 minutes, after which point the reaction was placed into an oil bath at 70 ºC to initiate the reaction. Once complete, the reaction was quenched in an ice bath followed by exposure to the atmosphere. The solution was precipitated into cold methanol or isopropanol and the product collected by filtration.

Poly(vinylbenzyl) chloride (P1)

^{1}H NMR (400 MHz, DMSO-d_6) δ: 7.18 (br, 2H, ortho-CH), 6.68 (br, 2H, meta-CH), 4.64 (br, 2H, CH_2), 1.89 (br, CH backbone), 1.64 (br, CH_2 backbone), 1.39 (br, RAFT-CH_2), 0.86 (br, RAFT-CH_3).

FT-IR (ATR): 2925 (w, C-H), 1723 (w, C=C), 1612, 1510, 1443, 1421, 1264, 1187, 1133, 1019, 824, 671, 638, 534

Poly(vinylbenzyl)chloride-alt-1-(3-(triisopropylsilyl)prop-2-ynyl)-1H-pyrrole-2,5-dione (P9)

^{1}H NMR (400 MHz, DMSO-d_6) δ: 7.17 (br, 2H, ortho-CH), 6.74 (br, 2H, meta-CH), 4.52 (br, 2H, CH_2), 4.10 (br, 2H, CH_2), 3.22 (br, CH backbone), 2.55(br, CH backbone), 2.08 (br, CH_2 backbone), 1.39 (br, RAFT-CH_2), 0.87 (br, RAFT-CH_3), 0.13 (s, 9H, TMS).

FT-IR (ATR): 2960 (w, C-H), 2185 (w, C≡C), 1776 (w, C=C), 1702 (s, C=O), 1512, 1419, 1393, 1342, 1249, 1177, 1113, 1006, 946, 841, 759, 676, 636

General procedure for the polymer azidation

The synthesis has been modified from previously reported method[10]. Briefly, the polymer was suspended in DMSO (300 % v/v) followed by the addition of NaN_3 (1.5 equiv per chloride) and stirred for 16 hours at 25 ºC. The resulting solution was diluted into water and extracted in EtOAc (3 x 50 mL). The organic layers were combined, dried over $MgSO_4$, filtered and reduced under negative pressure to give the polymer.

Poly(vinylbenzyl) azide (P2)

^{1}H NMR (400 MHz, DMSO-d_6) δ: 7.12 (br, 2H, ortho-CH), 6.70 (br, 2H, meta-CH), 4.38 (br, 2H, CH_2), 1.85 (br, CH backbone), 1.65 (br, CH_2 backbone), 1.41 (br, RAFT-CH_2), 0.88 (br, RAFT-CH_3).

FT-IR (ATR): 2924 (w, C-H), 2091 (s, N_3), 1758 (w, C=C), 1721, 1676, 1611, 1510, 1446, 1421, 1341, 1245, 1205, 1112, 1019, 877, 843, 805, 753, 703, 666, 556.

pVBAz-alt-TMS PMI (P10)

^{1}H NMR (400 MHz, Acetone-d_6) δ: 7.29 (br, 2H, ortho-CH), 6.74 (br, 2H, meta-CH), 4.66 (br, 2H, CH_2), 4.07 (br, 2H, CH_2), 2.70 (br, CH backbone), 2.07 (br, CH backbone), 1.65 (br, CH_2 backbone), 1.42 (br, RAFT-CH_2), 1.04 (br, RAFT-CH_3).

FT-IR (ATR): 2960 (w, C-H), 2183 (w, C≡C), 2091 (s, N_3), 1773 (w, C=C), 1700 (s, C=O), 1512, 1417, 1394, 1341, 1248, 1177, 1110, 1011, 950, 840, 759, 698, 642

General procedure for CuAAc click reaction

The polymer (1 equiv with respect to azide), alkyne sugar (1.5 equiv.), Me_6TREN (2 equiv) were suspended in DMSO (2 ml/g of solid) and degassed by the gentle bubbling of Ar gas for 30 minutes. CuBr was charged into a Schlenk tube and degassed by the gentle bubbling of Ar gas for 30 minutes. The degassed solution was cannulated into the Schlenk tube, with the solution and catalyst being stirred. The reaction was stirred at 25 ºC for 2 days. The resulting solution was diluted by water (v/v) and passed through Al_2O_3 (neutral). The resulting solution was dialyzed in the presence of cuprisorb© and then lyophilized to give the pure product.

Poly(vinylbenzyl) – graft – Glc (P3)

^{1}H NMR (400 MHz, D_2O) δ:8.18 (br, 1H, triazole-H), 6.97 (br, 4H, ortho-CH, meta-CH), 5.33 (br, 2H, C**H$_2$**), 4.99 (br, 2H, OC**H$_2$**), 3.55 (br, 5H Sugar-H), 1.30 (br, RAFT-C**H$_2$**), 0.89 (br, RAFT-C**H$_3$**).

FT-IR (ATR): 3332, (br, O-H), 2917 (w, C-H), 1222, 1018, 787, 557, 459

Poly(vinylbenzyl) – graft – Man (P4)

^{1}H NMR (400 MHz, D_2O) δ: 8.18 (br, 1H, triazole-H), 7.08 (br, 4H, ortho-CH, meta-CH), 5.56 (br, 2H, C**H$_2$**), 4.92 (br, 2H, OC**H$_2$**), 3.70 (br, 5H Sugar-H), 1.30 (br, RAFT-C**H$_2$**), 0.90 (br, RAFT-C**H$_3$**).

FT-IR (ATR): 3332, (br, O-H), 2946 (w, C-H), 1639, 1326, 1022, 788, 537

Poly(vinylbenzyl) – graft – Gal (P5)

^{1}H NMR (400 MHz, D_2O) δ: 8.11 (br, 1H, triazole-H), 6.96 (br, 4H, ortho-CH, meta-CH), 5.53 (br, 2H, C**H$_2$**), 4.50 (br, 2H, OC**H$_2$**), 3.70 (br, 5H Sugar-H), 1.22 (br, RAFT-C**H$_2$**), 0.81 (br, RAFT-C**H$_3$**).

FT-IR (ATR): 3348, (br, O-H), 2928 (w, C-H), 1611, 1512, 1425, 1345, 1223, 1041, 789, 540

Poly(vinylbenzyl) – graft – GlcNAc (P6)

^{1}H NMR (400 MHz, D_2O) δ: 8.12 (br, 1H, triazole-H), 7.66 (br, 1H, NH), 6.90 (br, 2H, ortho-CH), 6.40 (br, 2H, meta-CH), 5.47 (br, 2H, C**H$_2$**), 5.01 (br, 2H, OC**H$_2$**), 4.77 (br, 1H, CH Sugar-**H**), 4.64 (br, 2H, Sugar-**H**), 3.69 (br, 1H, C**H**), 3.48 (br, 2H, C**H$_2$**), 3.12 (br, 2H, Sugar-**H**), 1.67 (br, 3H, C**H$_3$**), 1.24 (br, RAFT-C**H$_2$**), 0.85 (br, RAFT-C**H$_3$**).

FT-IR (ATR): 3348, (br, O-H), 2928 (w, C-H), 1611, 1512, 1425, 1345, 1223, 1041, 789, 540

Poly(vinylbenzyl) – graft – GlcA (P7)

^{1}H NMR (400 MHz, D_2O) δ: 8.13 (br, 1H, triazole-H), 6.90 (br, 2H, ortho-CH), 6.37 (br, 2H, meta-CH), 5.39 (br, 2H, C**H$_2$**), 5.04 (br, 2H, OC**H$_2$**), 4.80 (br, 1H, CH Sugar-**H**), 4.66 (br, 2H, Sugar-**H**), 3.69 (br, 1H, C**H**), 3.48 (br, 2H, C**H$_2$**), 3.12 (br, 2H, Sugar-**H**), 1.24 (br, RAFT-C**H$_2$**), 0.85 (br, RAFT-C**H$_3$**).

FT-IR (ATR): 3356, (br, O-H), 2919 (w, C-H), 1740, 1605, 1512, 1423, 1289, 1221, 1043, 789, 544.

Poly(vinylbenzyl) – graft – GlcNAc-co-(vinylbenzyl)-graft-GlcA (P8)

^{1}H NMR (400 MHz, D_2O) δ: 8.18 (br, 1H, triazole-H), 7.05 (br, 4H, ortho-CH, meta-CH), 5.33 (br, 2H, C**H$_2$**), 3.74-3.55 (br, 4H, Sugar-**H**), 1.67 (br, 1.5H, C**H$_3$**)1.28 (br, RAFT-C**H$_2$**), 0.90 (br, RAFT-C**H$_3$**). FT-IR (ATR): 3268, (br, O-H), 2875 (w, C-H), 1608, 1546, 1419, 1345, 1214, 1037, 777, 532

General procedure for TMS deprotection of polymers

The polymer was suspended in DMSO (10% v/v) in water and placed within a 1 kDa dialysis membrane. The glycopolymer was dialyzed against K_2CO_3 solution followed by H_2O. The deprotected glycopolymer was freeze-dried to give the deprotected glycopolymer as a lyophilized powder.

Poly(vinylbenzyl) – graft – GlcNAc-alt-(vinylbenzyl)-graft-GlcA (P12)

^{1}H NMR (400 MHz, D_2O) δ: 7.97 (br, 1H, triazole-H), 6.92 (br, 4H, ortho-CH, meta-CH), 5.50 (br, 2H, C**H$_2$**), 4.29-3.33 (br, 14H, backbone, Sugar-**H**), 1.87 (br, 3H, C**H$_3$**)1.28 (br, RAFT-C**H$_2$**), 0.92 (br, RAFT-C**H$_3$**).

FT-IR (ATR): 3293, (br, O-H), 2934 (w, C-H), 1672 (C=O), 1462, 1405, 1324, 1257, 1196, 1127, 1052, 831, 798, 720, 596, 515

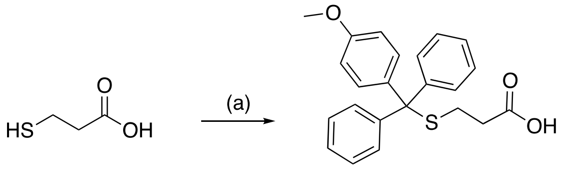

Scheme S3. Protection of mercaptopropionic acid. Reagents and condition: (a) MMT-Chloride, DIPEA, 1:1 DCM DMF

3-(((4-methoxyphenyl)diphenylmethyl)thio)propanoic acid

To a stirring solution of MMT-Chloride (3.5 g, 11.33 mmol) in 1:1 DCM/DMF (30 mL) and DIPEA (2.8 mL), the mercapto acid (0.82 mL, 9.4 mmol) was added dropwise. The reaction was allowed to stir overnight. The reaction mixture was concentrated and then suspended in H_2O (50 mL) which was washed with ether (3 x 50 mL). The organic layer was washed with brine (100 mL), dried over $MgSO_4$ and reduced under negative pressure. The oil was dried under high vacuum leaving a white powder (1.71 g, 4.5 mmol, 48%).

^{1}H NMR (400 MHz, $CDCl_3$) δ: 7.49 (d, 4H, 7.5 Hz, *m*-C**H**), 7.39 (d, 2H, 8.9 Hz, *o*-C**H)**, 7.35 (t, 4H, 7.1 Hz, *o*-C**H)**, 7.29 (d, 2H, 7.1 Hz, *p*-C**H)**, 6.89 (d, 2H, 8.9 Hz, *m*-C**H)**, 3.86 (s, CH_3), 2.54 (t, 5.1 Hz, 2H, SC$\mathbf{H_2}$), 2.34 (t, 5.1 Hz, 2H, C$\mathbf{H_2}$COOH).

^{13}C NMR (133 MHz, $CDCl_3$) δ: 177.52 (1C, **C**OOH), 158.23 (1C, H_3CO**C)**, 145.00 (1C, **C**C), 136.78, 130.87 (2C, o-**C**H), 129.58 (4C, *m*-**C**H), 128.04 (4C, o-**C**H), 126.79 (2C, *p*-**C**H), 113.32 (2C, *m*-**C**H), 66.56 (1H **C**S), 55.35 (1C, **C**H_3), 33.53 (1C, **C**H_2COOH), 26.72 (1C, S**C**H_2).

General procedure for microwave-assisted solid-phase peptide synthesis (SPSS)

The peptides were synthesised in an automated microwave peptide synthesizer (Liberty Blue, CEM) by solid-phase method on a Rink amide 4-methylbenzhydrylamine resin following standard 9-fluorenyl methoxycarbonyl (Fmoc) protocol. Couplings were done with the Fmoc-amino acids, HOBt and DIC in 4 times excess dissolved in DMF. The Fmoc group was deprotected with 20% piperidine in DMF (v/v). After each cycle, the resin was washed with DMF before the next cycle.

N-terminal thiol capping

After synthesis has been completed, the resin with bound peptide was removed from the synthesiser and 3-(((4-methoxyphenyl) diphenylmethyl)thio)propanoic acid was coupled to the peptide N-terminal using HOBt and DIC in 4 times excess. The mixture was shaken for 2 hours. After the capping cycle, the resin was subsequently washed with DMF followed by DCM and Kaiser test performed to confirm the coupling.

Thiol peptide cleavage

The peptide and acid-labile side groups were cleaved by incubating the resin with TFA/thioanisole/anisole/EDT (90:5:2.5:2.5) for 3 hours. The solution was collected, concentrated by reduced pressure and precipitated in cold ether. The resulting suspension was collected by centrifugation and decanting the supernatant, followed by lyophilisation of the resulting in the crude peptide.

Peptide characterization and purification

The crude peptides were passed through a 0.2 µm filter and then purified on an AutoPurification System (Waters) using a preparative reverse-phase C18 column (XBridge, 130 Å, 5 µm, 30×150 mm, Waters) with a gradient running from 98:2 to 0:100 of water/acetonitrile supplemented with 0.1% TFA over 30 minutes at 20 mL/min. The fractions were detected by the SQ Mass Detector (Waters). These fractions were combined, reduced under negative pressure and then lyophilised to produce a white powder.

Supplementary Characterisation

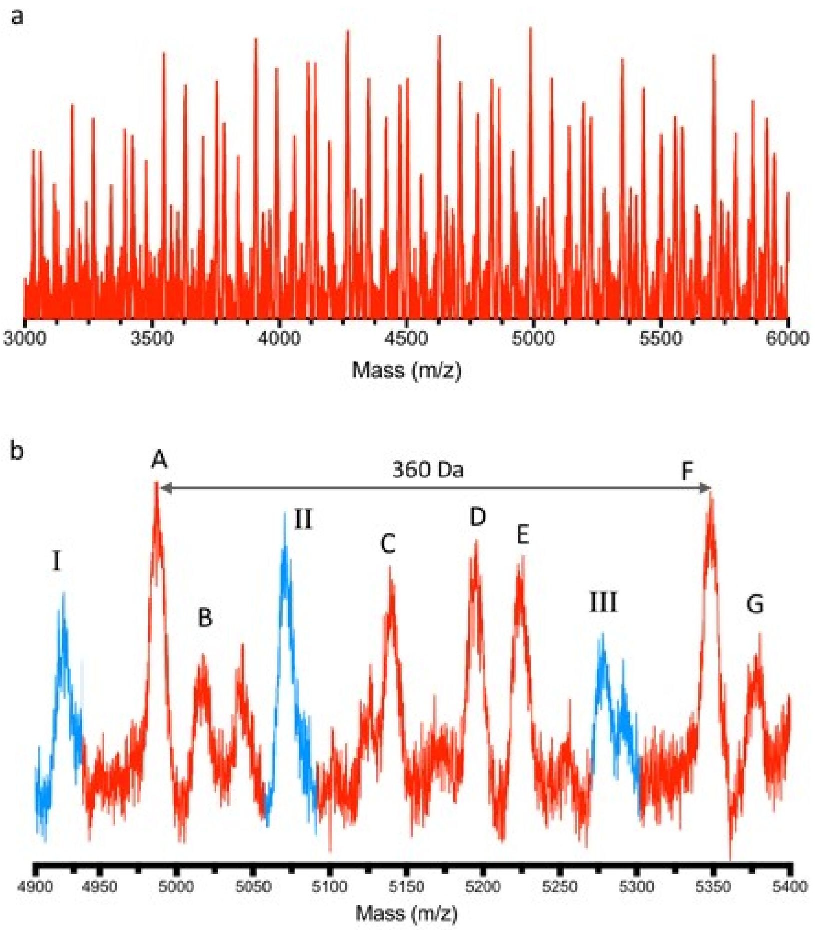

Figure S1. Low-resolution MALDI-ToF MS for P_9 between (a) 3000 – 6000 m/z showing multiple distributions with (b) a zoom between 4900 – 5400 m/z to look at a single repeat and the smaller distributions assigned (Table S1) between the major distribution highlighted by the arrow, with those polymers initiated with the initiator rather than the RAFT agent highlighted in blue.

Table S1. Inference for peaks between 4900 – 5400 m/z for P_9.

Peaks		Inference	Mass (m/z)	
RAFT	Initiator		Theoretical	Actual
-	I	($PVBC_{12}$-alt-TMS PMI_{13})	4918	4918
A	-		4972	4987
B	-	($PVBC_{12}$-alt-TMS PMI_{12}/TMS PMA+OMe)	5003	5018
-	II	($PVBC_{13}$-alt-TMS PMI_{13})	5071	5070
C	-		5126	5139
D	-	($PVBC_{12}$-alt-TMS PMI_{14})	5182	5195
E	-	($PVBC_{12}$-alt-TMS PMI_{13}/TMS PMA+OMe)	5213	5226
-	II	($PVBC_{13}$-alt-TMS PMI_{14})	5293	5278
F	-		5335	5349
G	-	($PVBC_{13}$-alt-TMS PMI_{13}/TMS PMA+OMe)	5366	5380

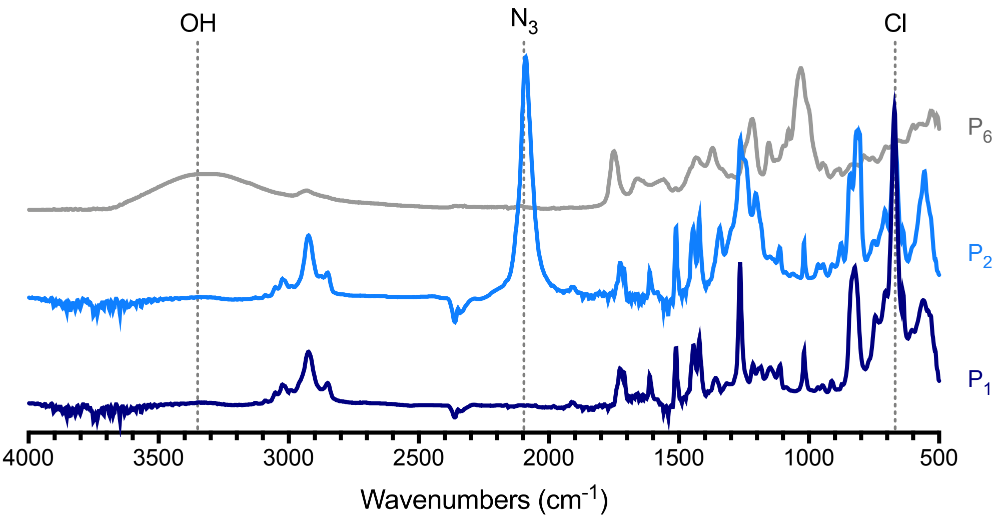

Figure S2. Offset FT-IR spectra to track the formation of the pendant azide at 2100 cm^{-1} of P_2 (light blue) from the chloride of P_1 (blue) followed by the consumption of the azide in the CuAAC to form the glycopolymer (pVB-GlcNAc, P_6)

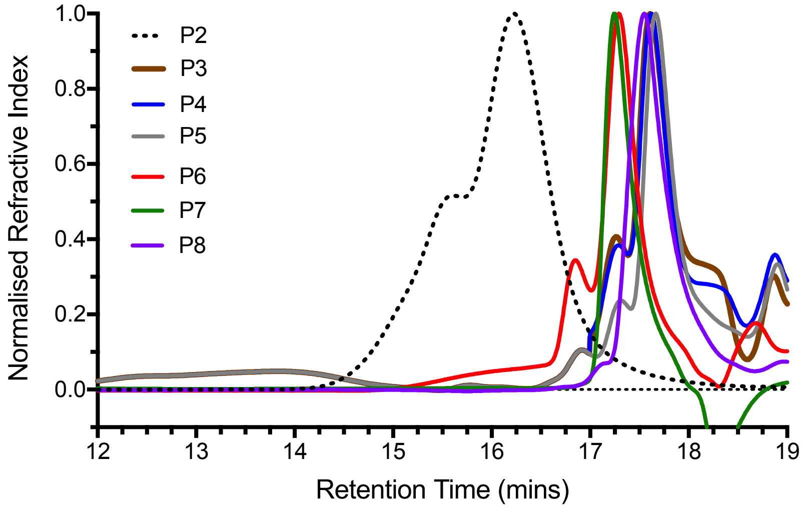

Figure S3. GPC traces of glycohomopolymers and the statistical glycocopolymer formed by CuAAc reaction with sugar alkynes with the parent polymer, P_2 (top).

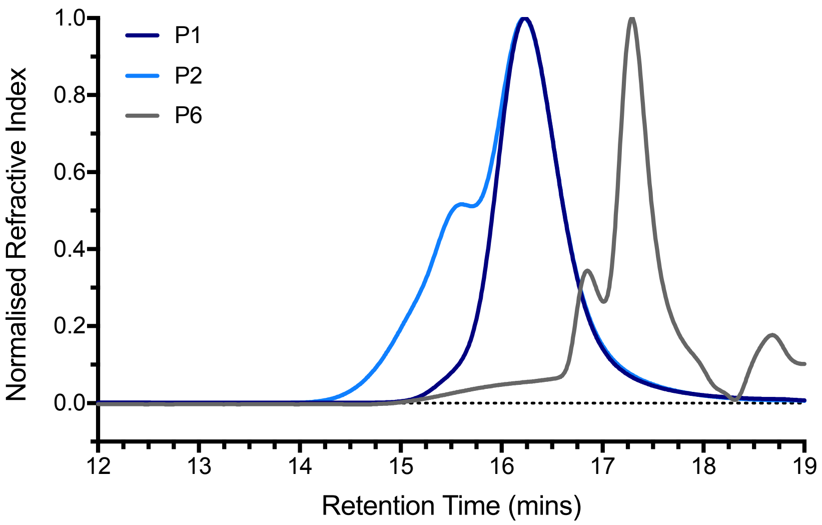

Figure S4. Example of GPC traces for the formation of a glycopolymer (pVB-GlcNAc, P_6) from the polymerisation of VBC to form P_1 (blue) through the conversion of the chloride to azide, P_2 (light blue) to the glycopolymer, P_6 (grey) by CuAAc.

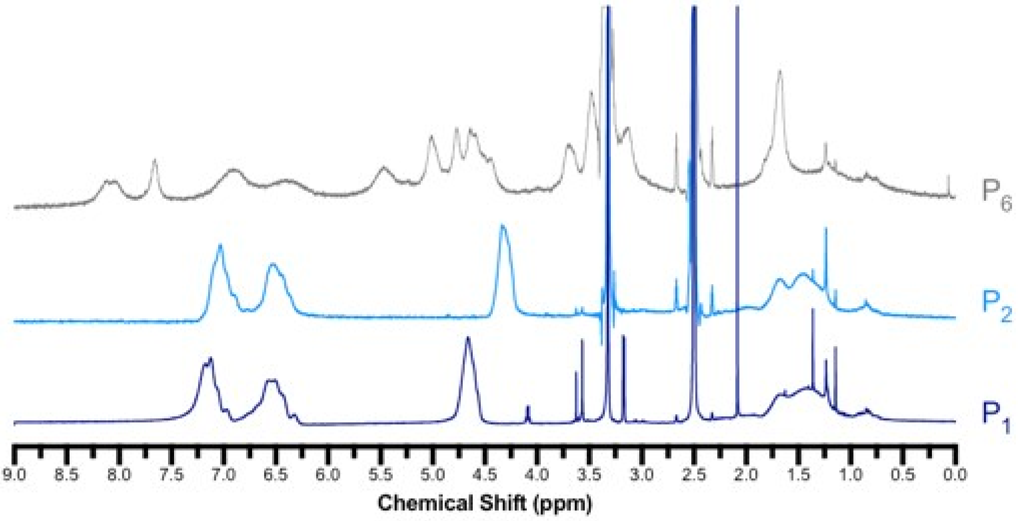

Figure S5. Example of NMR spectra (offset) to trace the formation of a glycopolymer (pVB-GlcNAc, P_6) the pendant group from pVBC (P_7) via PVBAz (P_2) showing the shift of the CH_2 at 4.6 ppm (P_1) to 4.3 ppm (P_2) as well as the introduction of the triazole peak at 8.1 ppm and sugar peaks between 3.0 – 5.0 ppm (P_6).

Table S2. GPC characterisation after azidation of pVBC free radical polymer.

**Entry**	**Polymer**	**Temp (°C)**	**$M_{n, GPC}$ (g/mol)[a]**	**Đ[a]**
**Parent Polymer**	pVBC	-	38000	1.95
**P1**	pVBAz	25	41000	1.76
**P2**	pVBAz	50	40000	1.80

[a] Determined by GPC using NH_4BF_4 (5 mM) in DMF with PS Calibration

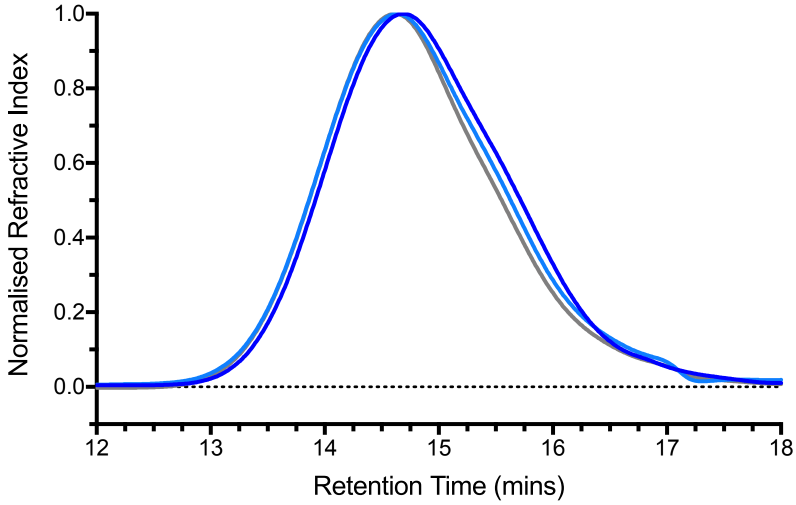

Figure S6. GPC traces of free radical polymerised pVBC with the conversion to PVBAz at 50 ºC and 25 ºC.

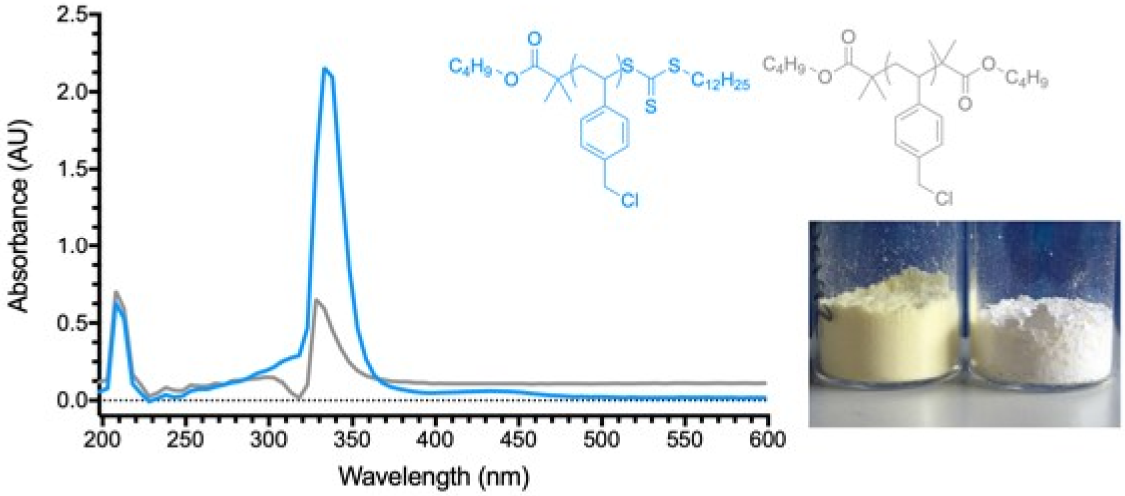

Figure S7. UV-Vis traces of incomplete radical-induced RAFT reduction of pVBC before (blue) and after two rounds of RAFT cleavage (light blue) 1 mg/mL in CH_2Cl_2 with an image insert showing the colour change of the polymer from yellow with the RAFT agent on the omega terminal and off-white with the initiator on the omega terminal.

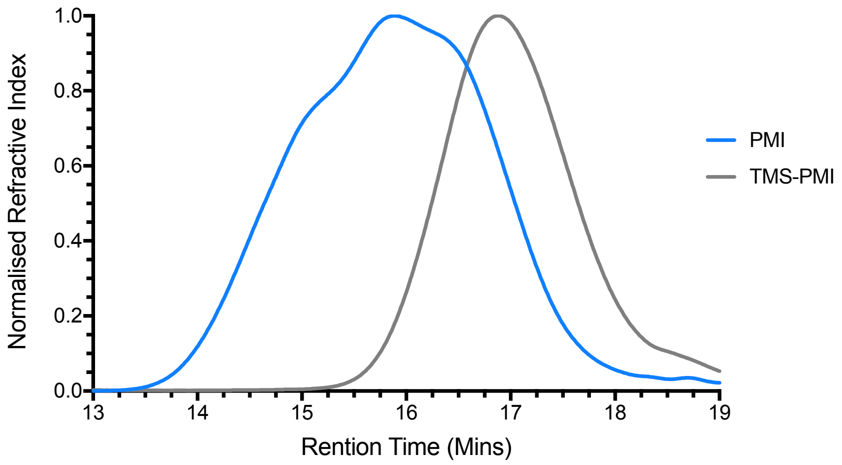

Figure S8. GPC traces of TMS protected alkyne PMI compared to unprotected alkyne on PMI.

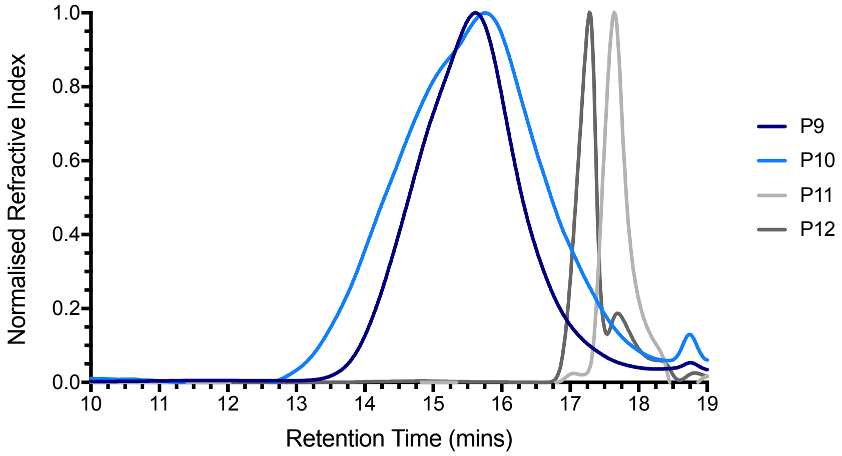

Figure S9. GPC traces of alternating glycopolymer (P12) formed by the polymerisation of P10, azidation P10, followed by the clicking of the first monosaccharide GlcNAc (P11) and removal of TMS to facilitate the clicking of the second monosaccharide GlcA (P12).

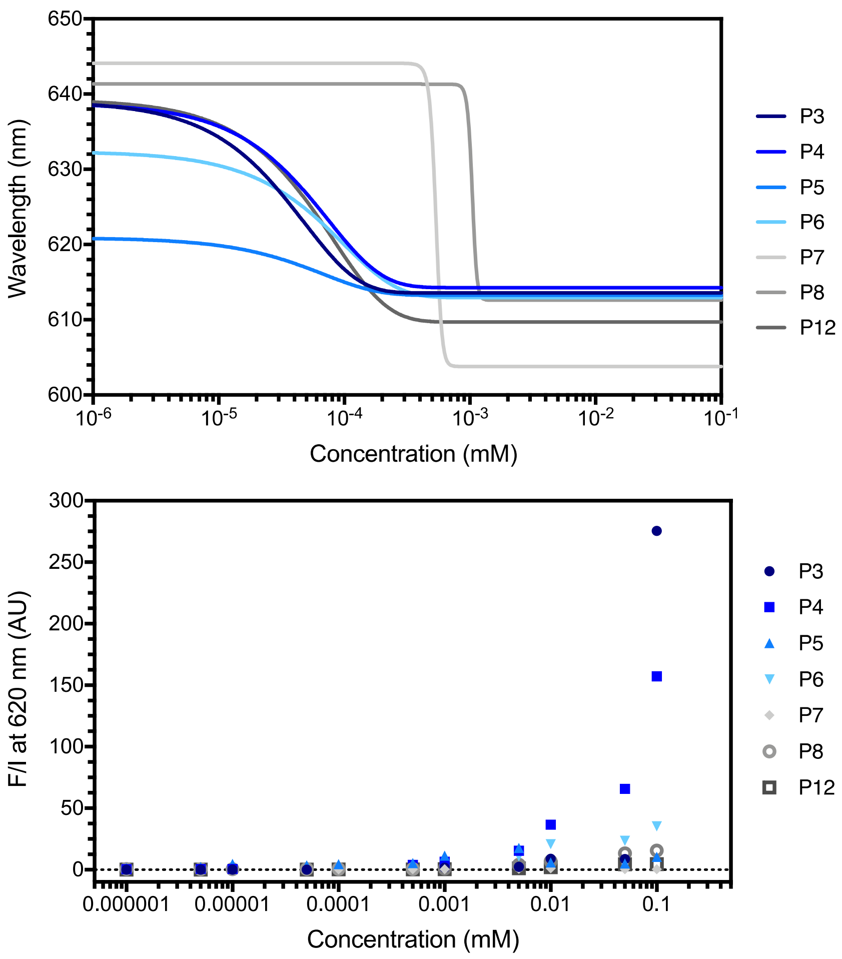

Figure S10. Nile Red fluorescence for critical aggregation concentration determination by (top) looking at the change in fluorescent maxima as a function of concentration as well as (bottom) fluorescence upon the blue shift of the fluorescence maxima.

Table S3. Zeta-potential and CAC of glycopolymers.

**Polymer**		**Type**	**CAC /M[a]**	**ζ /mV[a]**
P3	pVB-Glc$_{56}$	Glycohomopolymer	5.5 x 10^{-7}	-1.55 ± 0.76
P4	pVB-Man$_{56}$	Glycohomopolymer	6.3 x 10^{-7}	1.59 ± 1.93
P5	pVB-Gal$_{56}$	Glycohomopolymer	< 1 x 10^{-9}	-0.85 ± 1.73
P6	pVB-GlcNAc$_{56}$	Glycohomopolymer	8.5 x 10^{-8}	+ 0.18 ± 0.53
P7	pVB-GlcA$_{56}$	Glycohomopolymer	3.2 x 10^{-6}	- 4.67 ± 0.67
P8	pVB-GlcA$_{23}$-co-VB-GlcNAc$_{23}$	Statistical Glycocopolymer	2.1 x 10^{-6}	- 3.70 ± 1.21
P12	pVB-GlcNAc$_{24}$-alt-PMI-GlcA$_{24}$	Alternating Glycocopolymer	8.3 x 10^{-7}	- 9.44 ± 0.71

[a] H_2O at pH = 7. n.m.

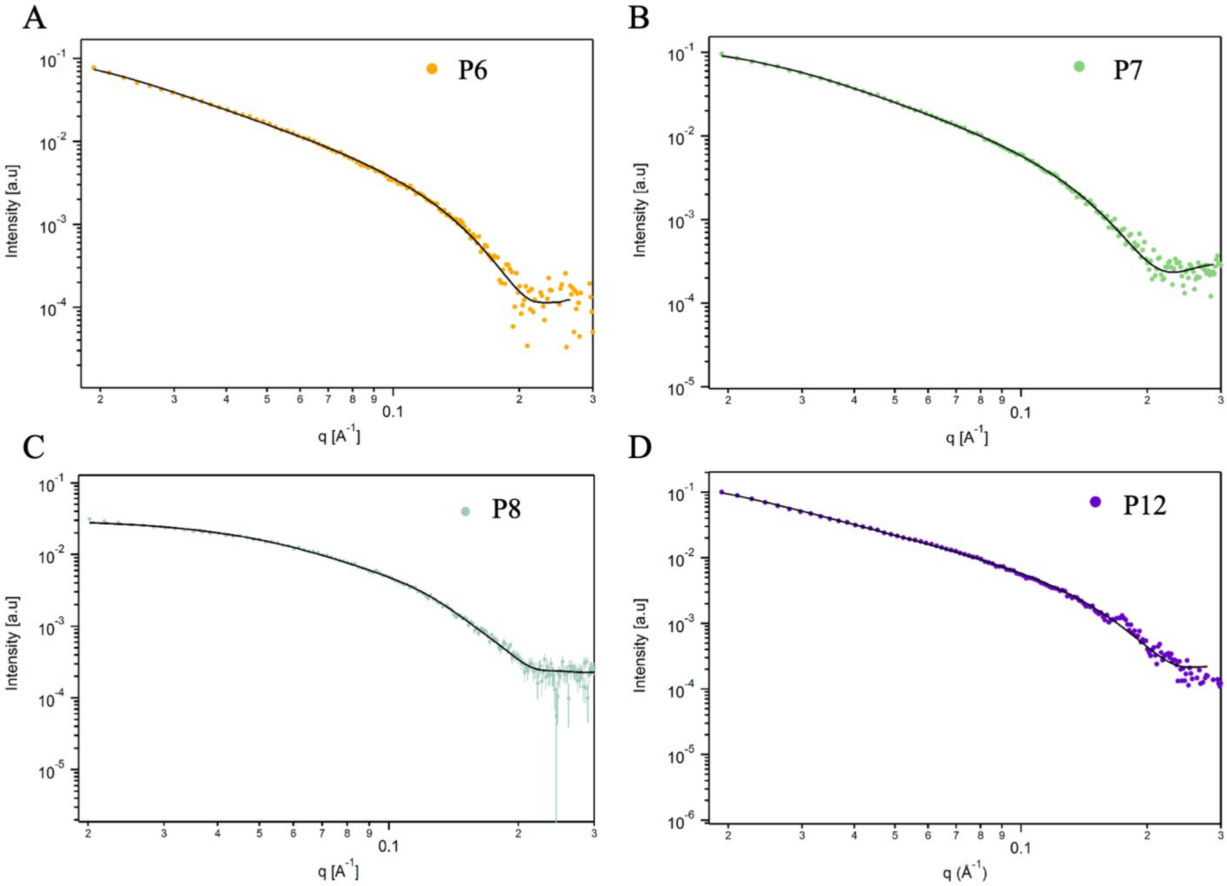

Figure S11. SAXS data of the glycopolymers fitted to a model shown in the black line.

Table S4. Parameters from the SAXS fitted models of the glycopolymers.

Glycopolymer	Kuhn length (Å)	Radius (Å)	Contour Length (Å)
P6	51	16	700
P7	49	16	660
P8	50	15	570
P12	52	15	700

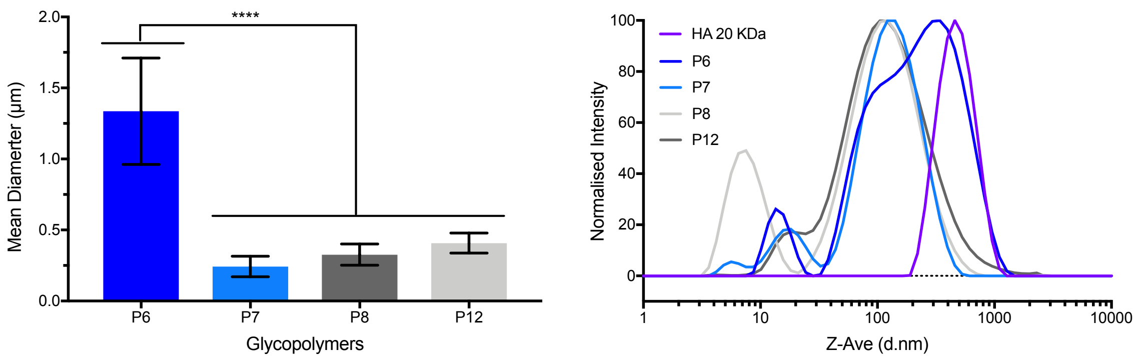

Figure S12. Glycopolymer mean size distribution of the aggregates obtained by ImageJ analysis of TEM images (left) and DLS on the glycopolymers at 0.1 mM at pH 7. Analysed by 2one-way ANOVA analysis, where * significant at P < 0.0332, ** significant at P < 0.0021, *** significant at P < 0.0002 and **** significant at P < 0.0001.

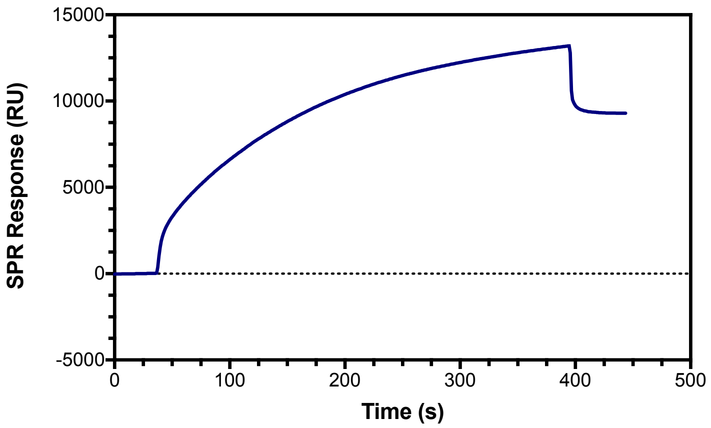

Figure S13. SPR sensorgram of CD44 (0.025 mg/ml) immobilization via a standard amine coupling protocol onto a CM5 sensor chip over 6 min at 25 °C at a flow rate of 20 µL/min.

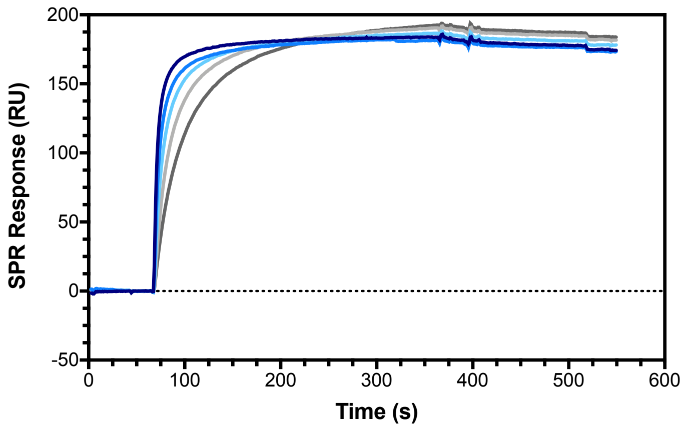

Figure S14. SPR sensorgrams of CD44 with HA 20 kDa showing saturation between the highest concentration (2000 nM) to the lowest concentration (125 nM).

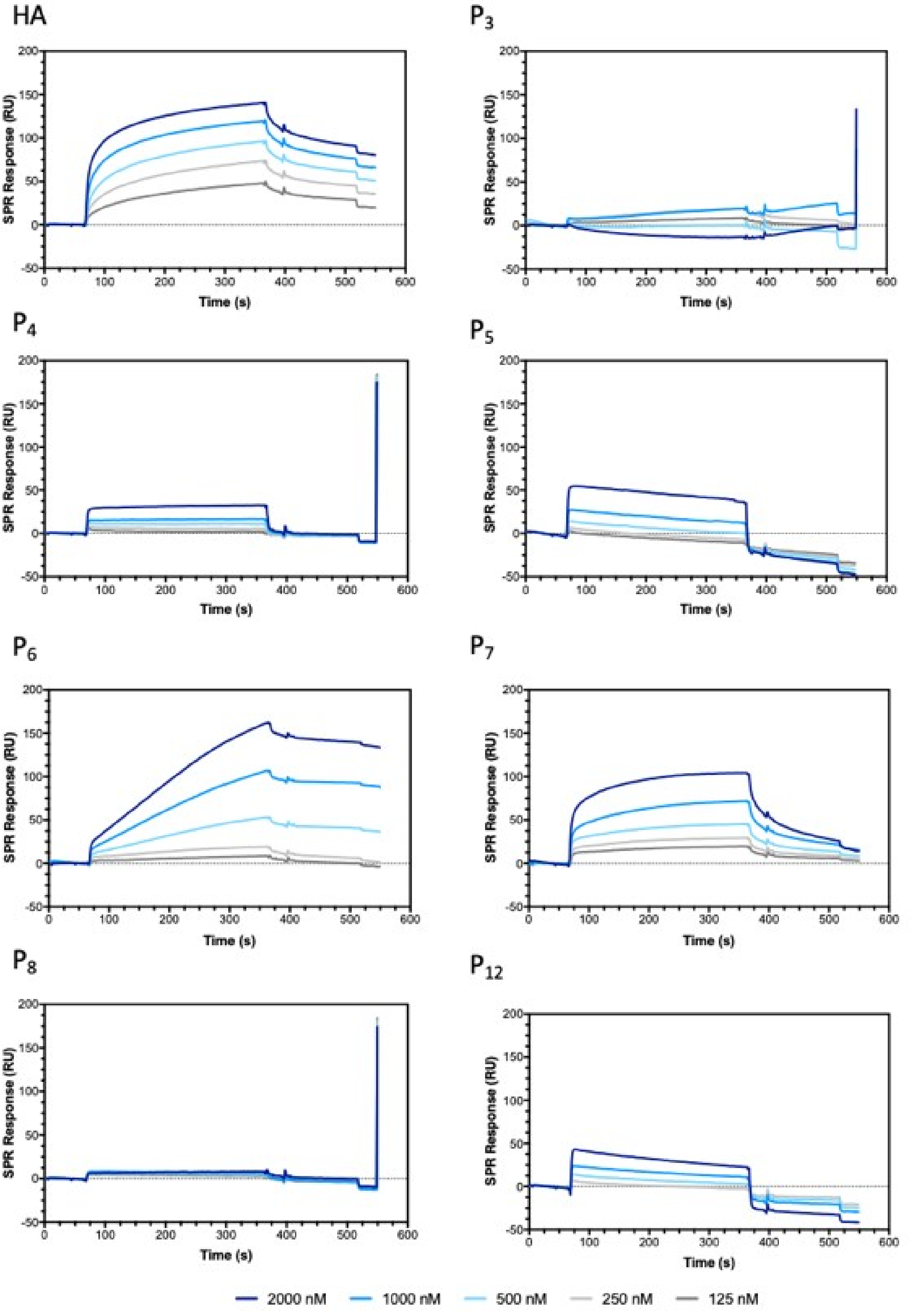

Figure S15. SPR sensorgrams for CD44 with the various polymers and HA (5 kDa) at 25 °C at a flow rate of 10 µL/min

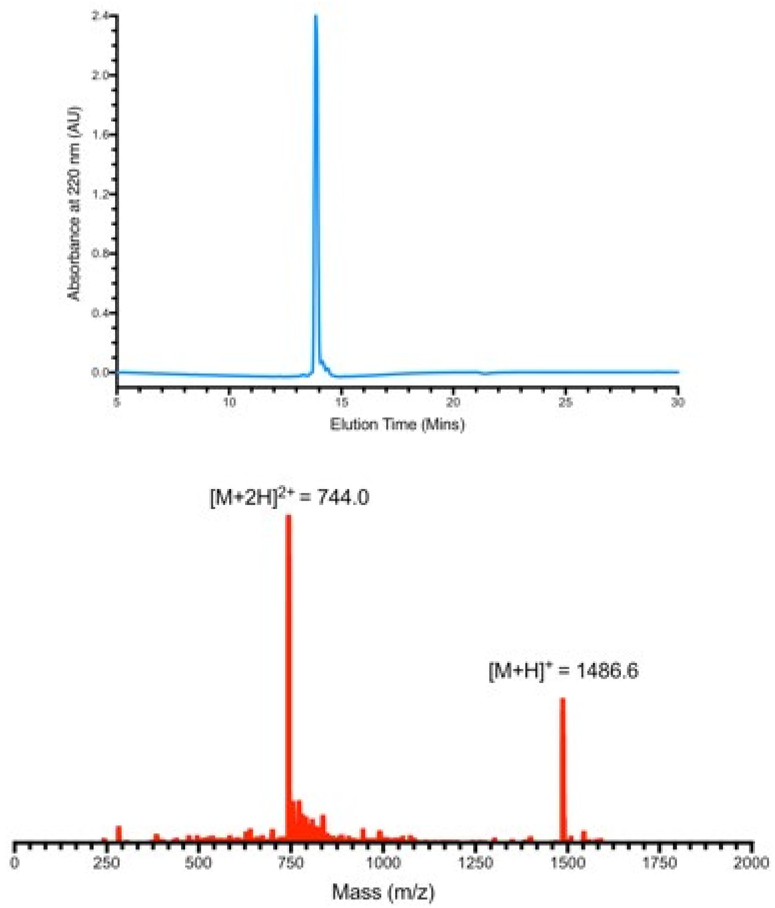

Figure S16. Characterisation of purified HS-GAHWQFNALTVR-CONH$_2$ peptide by analytical HPLC (top) and) ESI-MS (bottom showing a single peak and the observed masses, respectively. Expected m/z: 1486.7.

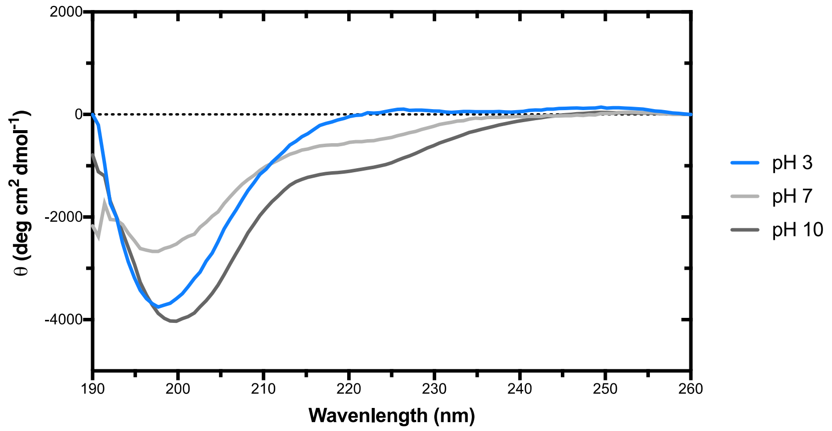

Figure S17. Circular dichroism spectra of acetylated (Ac-GAHWQFNALTVR-CONH$_2$, non-thiolated) Pep-1 at 110 µM in water at various pHs, showing a random coil.

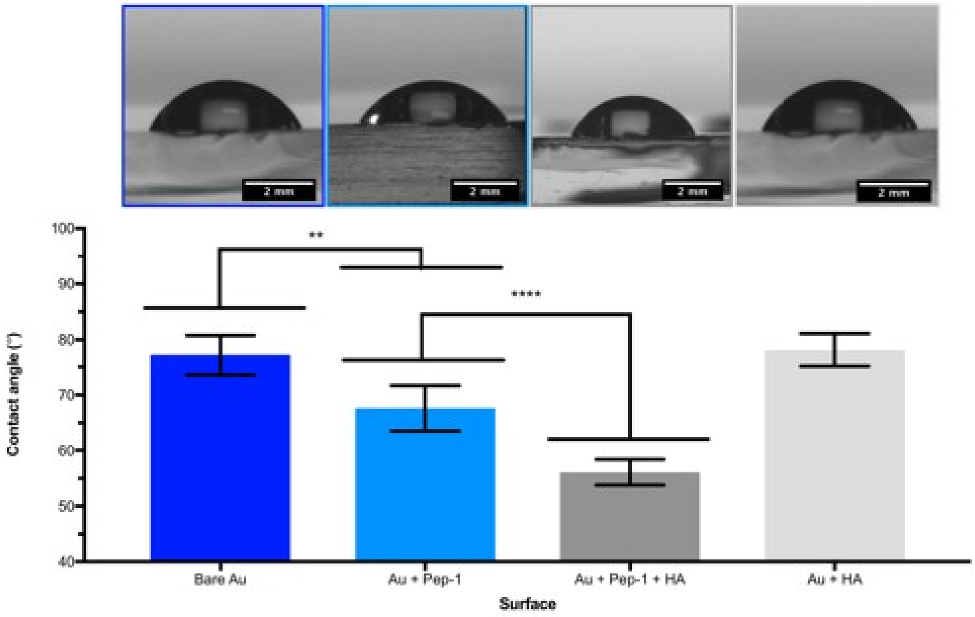

Figure S18. Contact angle of gold-coated coverslips, before and after immersion is an ethanoic solution of thiolated Pep-1 followed by the incubation with 1.5 MDa HA. Analysed by 2one-way ANOVA analysis, where * significant at P < 0.0332, ** significant at P < 0.0021, *** significant at P < 0.0002 and **** significant at P < 0.0001.

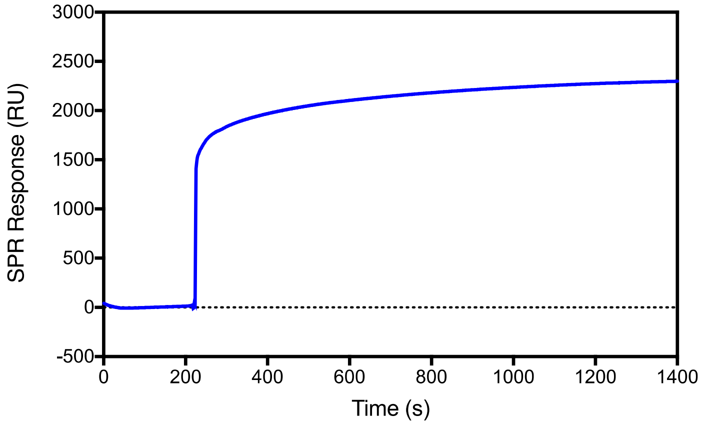

Figure S19. SPR sensorgram of Pep-1 adsorption onto the gold surface.

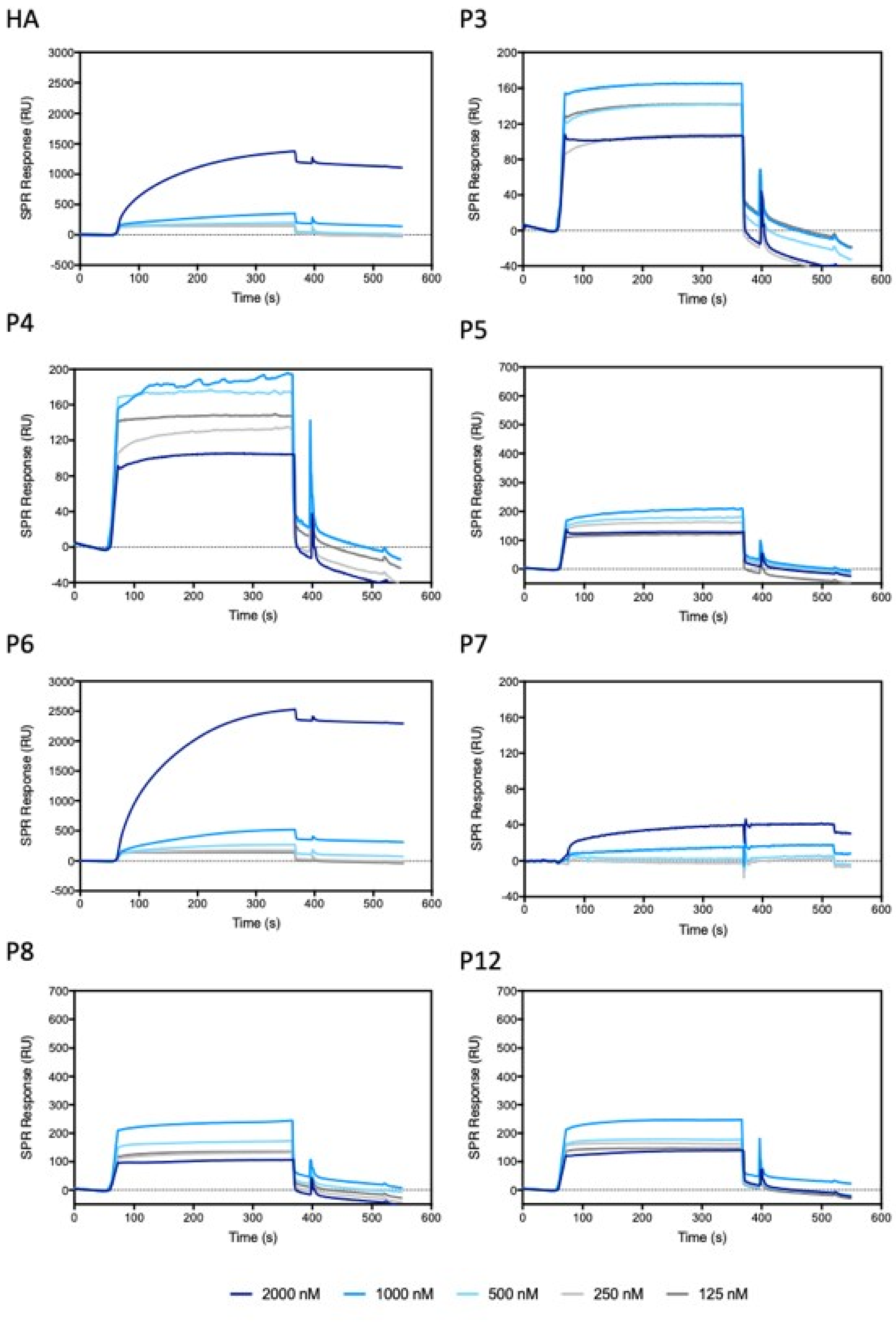

Figure S20. SPR sensorgrams for Pep-1 with the various polymers and HA (5 kDa) at 25 °C at a flow rate of 10 µL/min.

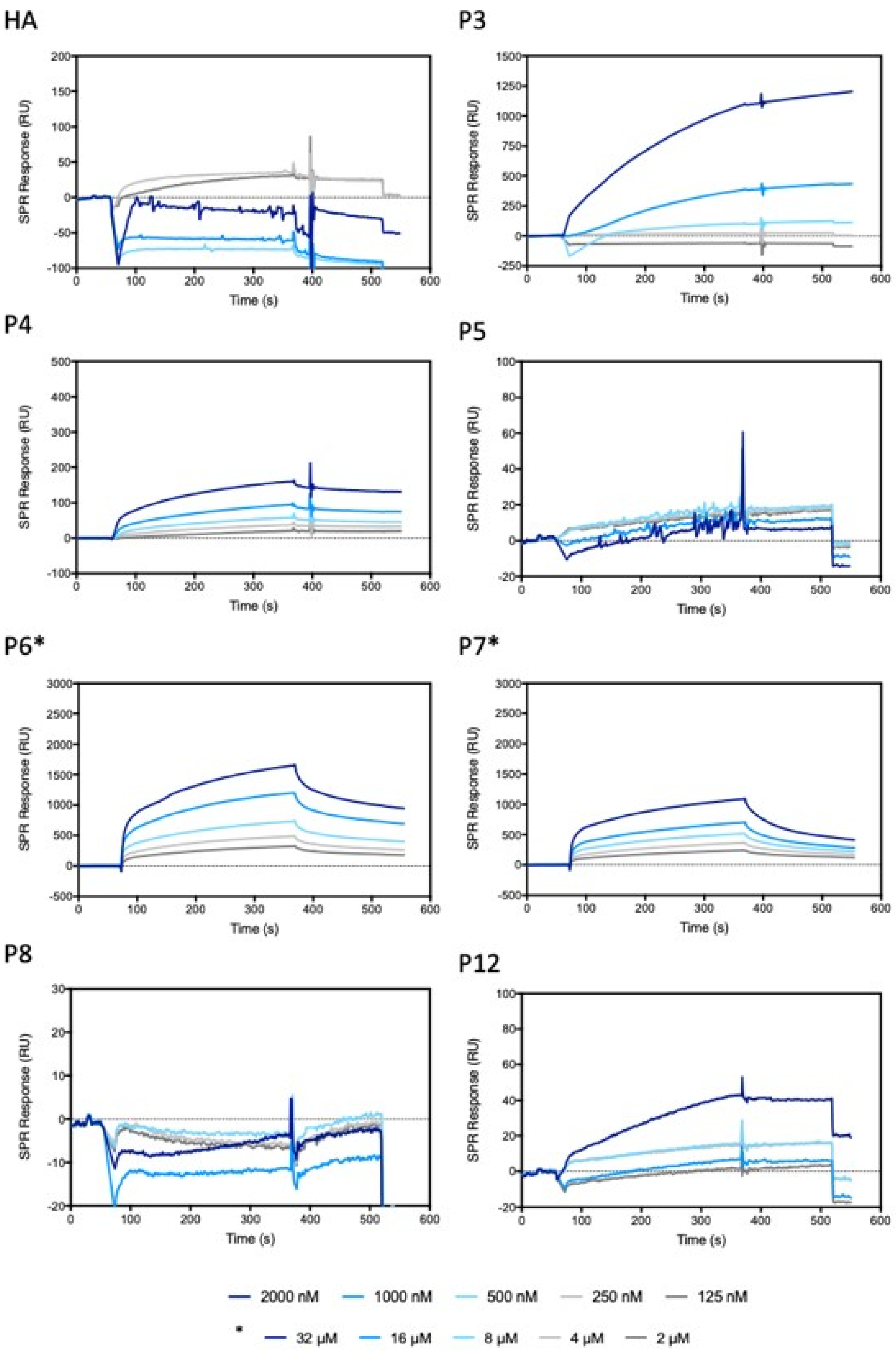

Figure S21. SPR sensorgrams for DC-SIGN with the various polymers and HA (5 kDa) at 25 °C at a flow rate of 10 µL/min. An increased concentration range was used for P_6 and P_7 denoted by *.

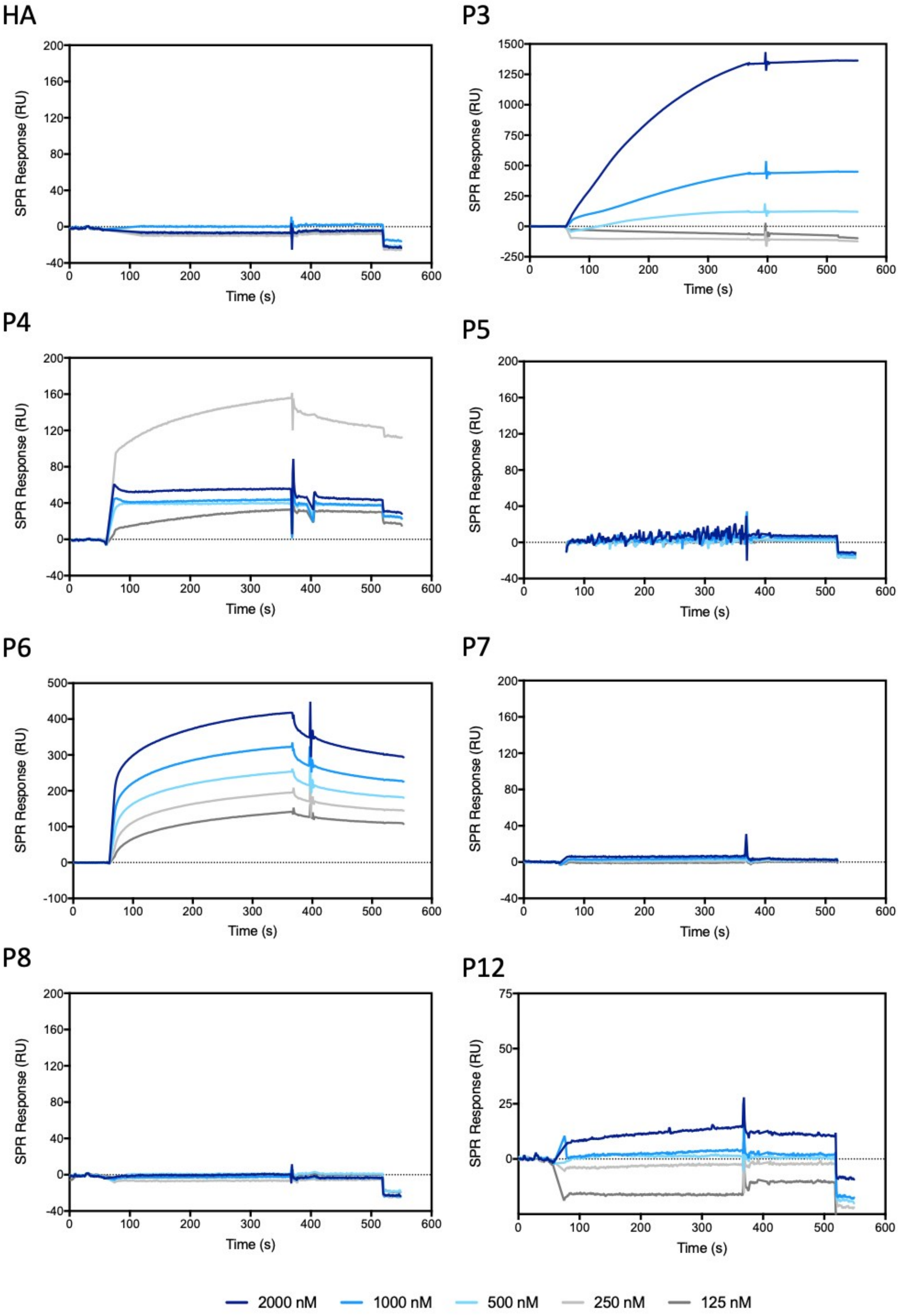

Figure S22. SPR sensorgrams for MBL with the various polymers and HA (5 kDa) at 25 °C at a flow rate of 10 µL/min

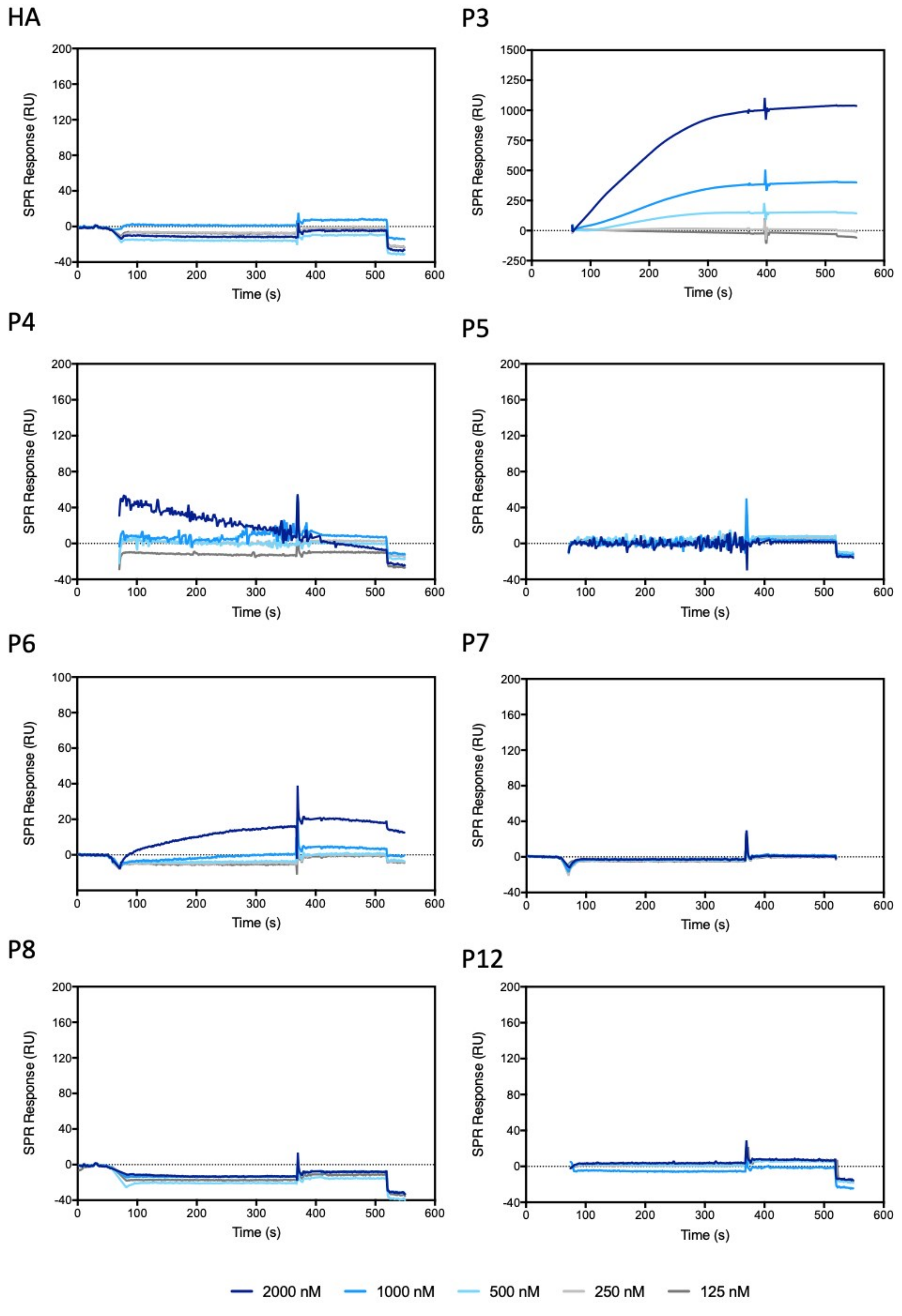

Figure S23. SPR sensorgrams for SP-D with the various polymers and HA (5 kDa) at 25 °C at a flow rate of 10 µL/min.

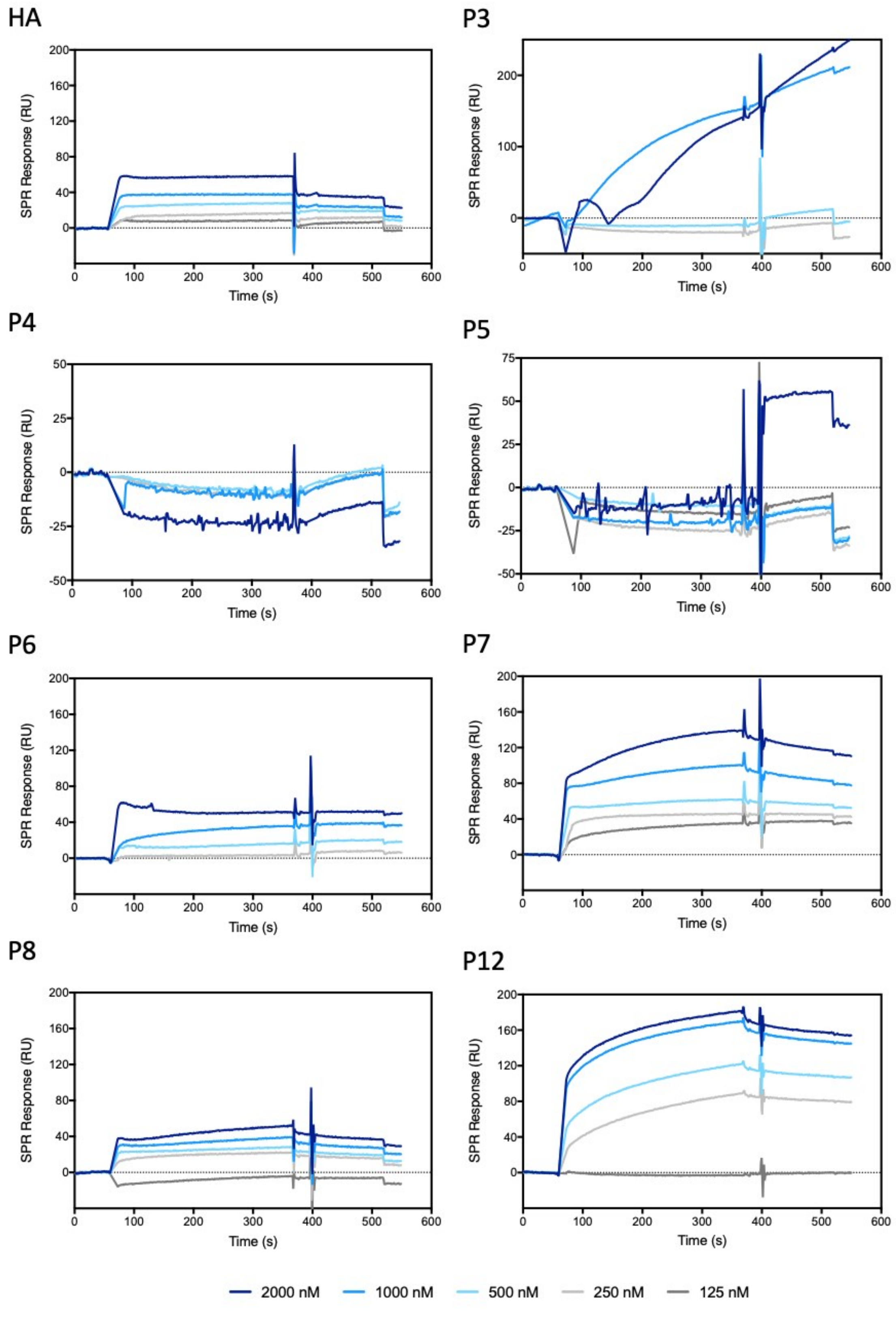

Figure S24. SPR sensorgrams for Dectin-1 with the various polymers and HA (5 kDa) at 25 °C at a flow rate of 10 µL/min.

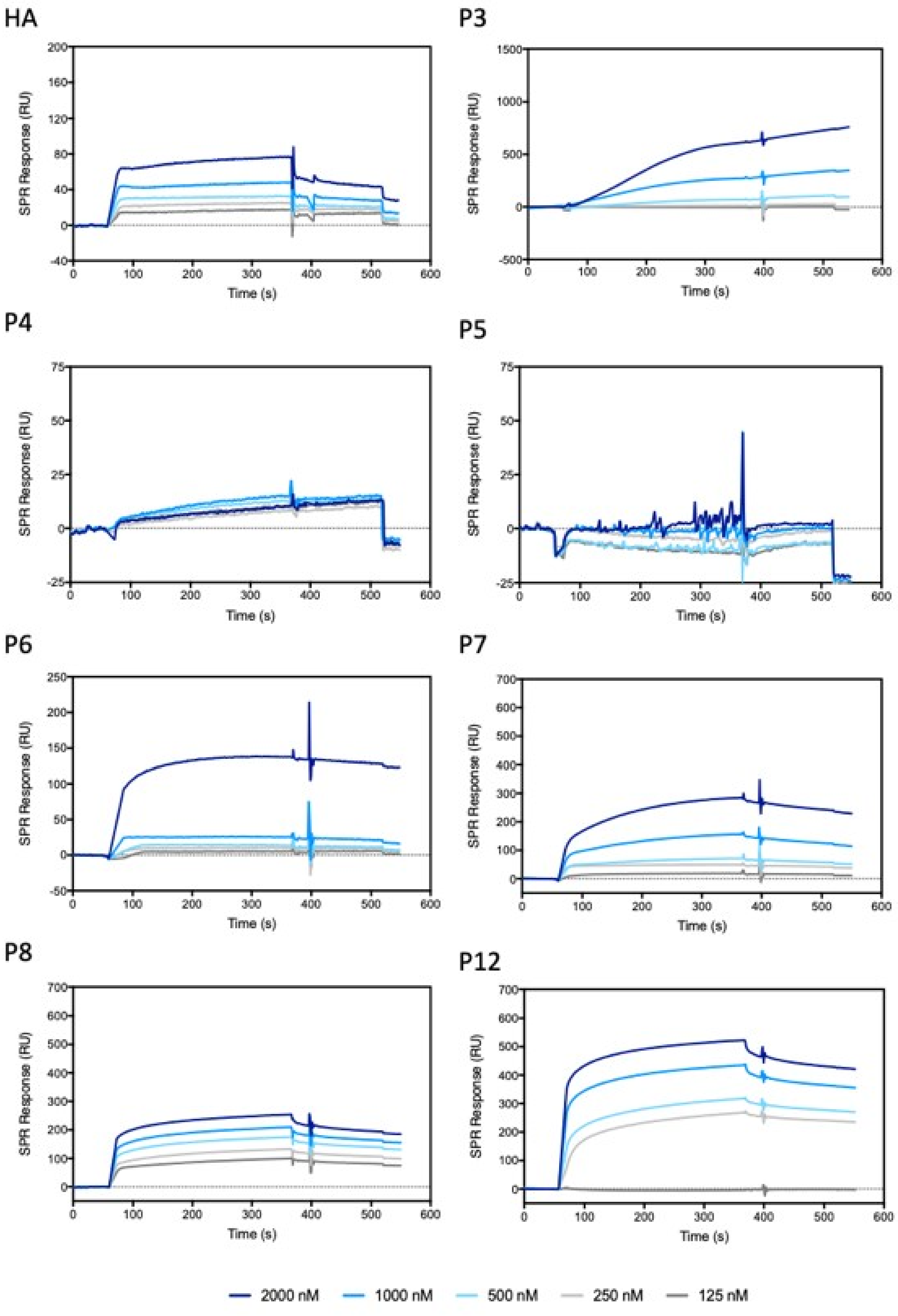

Figure S25. SPR sensorgrams for DEC-205 with the various polymers and HA (5 kDa) at 25 °C at a flow rate of 10 µL/min.

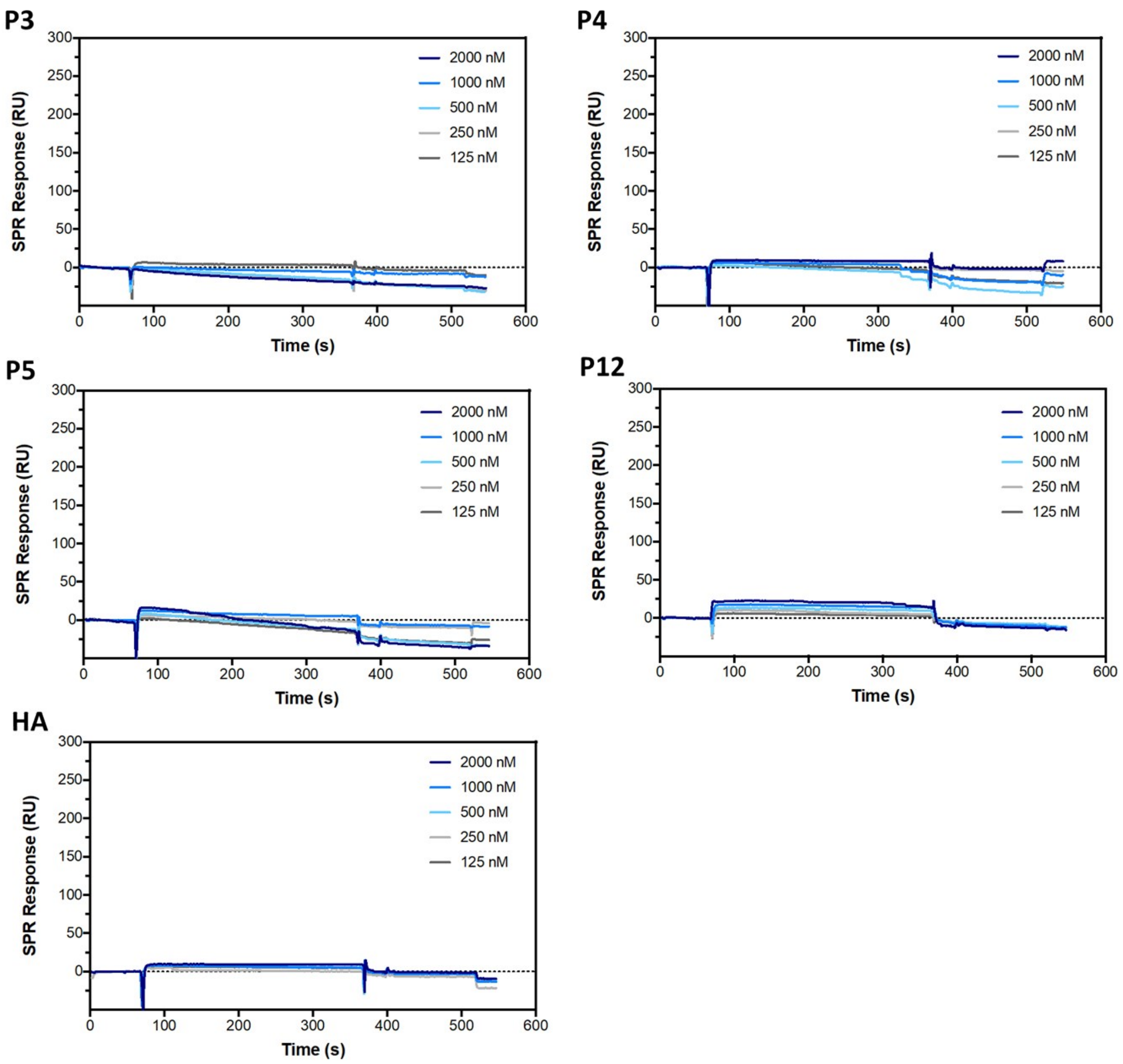

Figure S26. SPR sensorgrams for hyaluronidase with various polymers and HA (5 kDa) at 25 °C at a flow rate of 10 µL/min.

Table S5. Summarised SPR binding results for the HA and glycopolymers against an array of receptors.

	CD44		Pep-1		Dectin-1		DEC-205	
**Polymer**	RU_{Max}	K_D (M)	RU_{Max}	K_D (M)	RU_{Max}	K_D (M)	RU_{Max}	K_D (M)
**HA***	140	1.51×10^{-7}	1379	9.70×10^{-6}	58	1.57×10^{-7}	77	1.9×10^{-7}
**P3**	n.s.	n.s.	165	n.s.	157	n.s.	635	9.1×10^{-8}
**P4**	n.s.	n.s.	193	n.s.	0	n.s.	15	n.s.
**P5**	n.s.	n.s.	208	n.s.	138	n.s.	0	n.s.
**P6**	170	5.70×10^{-7}	2490	3.44×10^{-7}	50	n.s.	523	1.0×10^{-7}
**P7**	105	9.23×10^{-7}	40	n.s.	125	2.00×10^{-12}	303	1.7×10^{-8}
**P8**	n.s.	n.s.	245	n.s.	52	6.30×10^{-10}	254	9.1×10^{-10}
**P12**	n.s.	n.s.	247	n.s.	186	4.20×10^{-10}	523	1.3×10^{-11}

n.s. = non-specific binding. *HA 5 kDa for CD44, whilst it is maintained at 20 kDa for the other receptors.

Table S6. Summarised SPR binding results for glycopolymers binding to hyaluronidase.

	k_a (1/Ms)	k_d (1/s)	K_A (1/M)	K_D (M)	RU_{Max}
**Heparin**	1.51×10^{2}	1.53×10^{-4}	9.86×10^{5}	1.01×10^{-6}	220
**P6**	1.79×10^{3}	8.35×10^{-4}	2.14×10^{6}	4.66×10^{-7}	430
**P7**	5.04×10^{3}	3.39×10^{-3}	1.49×10^{6}	6.72×10^{-7}	275
**P8**	1.62×10	6.95×10^{-4}	2.33×10^{4}	4.29×10^{-5}	50

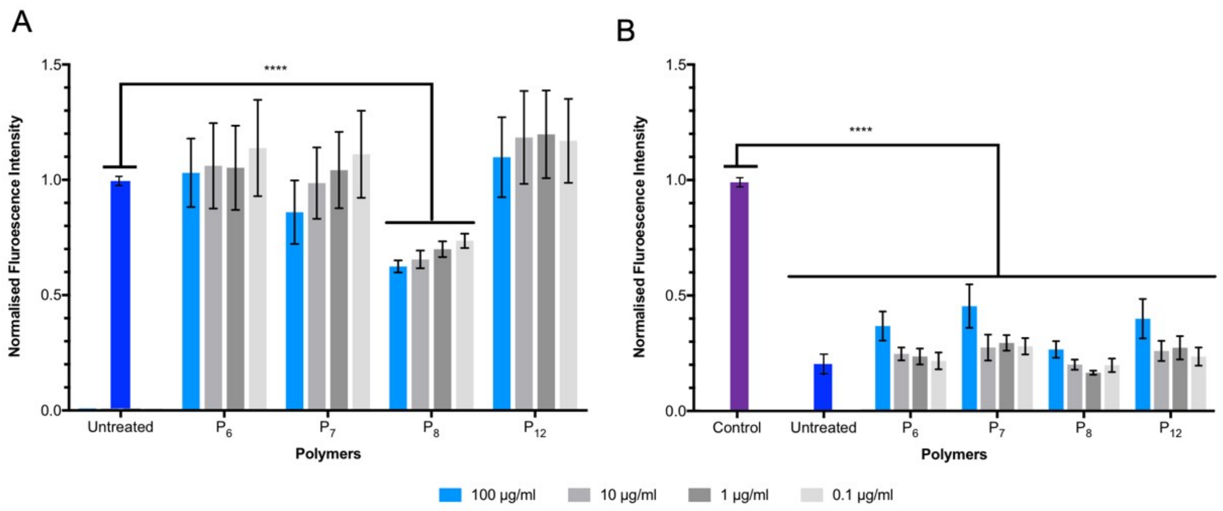

Figure S27. Viability of Luc4 cells cultured in media supplemented with P6, P7, P8 and P12 at four different concentrations (0.1, 1, 10, 100 µg/mL). Results were normalised fluorescence intensity of (A) untreated cells (live control) and (B) methanol treated cells (dead control). Analysed by 2way ANOVA analysis, **** significant at P < 0.0001.

References

1. J. Ilavsky and P. R. Jemian, *Journal of Applied Crystallography*, 2009, **42**, 347-353.
2. S. R. Kline, *Journal of Applied Crystallography*, 2006, **39**, 895-900.
3. J. S. Pedersen and P. Schurtenberger, *Macromolecules*, 1996, **29**, 7602-7612.
4. W.-R. Chen, P. D. Butler and L. J. Magid, *Langmuir*, 2006, **22**, 6539-6548.
5. B. Roy and B. Mukhopadhyay, *Tetrahedron Letters*, 2007, **48**, 3783-3787.
6. C. Loukou, M. Tosin, H. Mueller-Buriz and P. V. Murphy, *Carbohydrate Research*, 2007, **342**, 1953-1959.
7. H. R. Pfaendler and V. Weimar, *Synthesis*, 1996, **1996**, 1345-1349.
8. B. V. Schmidt, N. Fechler, J. Falkenhagen and J. F. Lutz, *Nat Chem*, 2011, **3**, 234-238.
9. J. Moraes, K. Ohno, G. Gody, T. Maschmeyer and S. Perrier, *Beilstein J Org Chem*, 2013, **9**, 1226-1234.
10. W.-H. Ting, S. A. Dai, Y.-F. Shih, I. K. Yang, W.-C. Su and R.-J. Jeng, *Polymer*, 2008, **49**, 1497-1505.
